# The Polo-like kinase 1 inhibitor onvansertib represents a relevant treatment for head and neck squamous cell carcinoma resistant to cisplatin and radiotherapy

**DOI:** 10.7150/thno.61711

**Published:** 2021-09-21

**Authors:** Anais Hagege, Damien Ambrosetti, Julien Boyer, Alexandre Bozec, Jérôme Doyen, Emmanuel Chamorey, Xingkang He, Isabelle Bourget, Julie Rousset, Esma Saada, Olivia Rastoin, Julien Parola, Frederic Luciano, Yihai Cao, Gilles Pagès, Maeva Dufies

**Affiliations:** 1 University Côte d'Azur, Institute for Research on Cancer and Aging of Nice (IRCAN), CNRS UMR 7284; INSERM U1081, Centre Antoine Lacassagne, 06189 Nice, France.; 2 LIA ROPSE, Laboratoire International Associé Université Côte d'Azur - Centre Scientifique de Monaco.; 3 University Côte d'Azur, Centre Hospitalier Universitaire (CHU) de Nice, Hôpital Pasteur, Central laboratory of Pathology, 06000 Nice, France.; 4 Centre Antoine Lacassagne, 06189 Nice, France.; 5 Department of Microbiology, Tumor and Cell Biology, Karolinska Institutet, SE-171 77 Stockholm, Sweden.; 6 Centre Scientifique de Monaco, Biomedical Department, 8 quai Antoine Premier, 98 000 Monaco, Principality of Monaco.

**Keywords:** Plk1, Head and neck squamous cell carcinoma (HNSCC), cisplatin resistance, radiation resistance, onvansertib

## Abstract

**Rationale:** Head and neck squamous cell carcinoma (HNSCC) represent the 4^th^ most aggressive cancer. 50% of patients relapse to the current treatments combining surgery, radiotherapy and cisplatin and die two years after the diagnosis. Elevated expression of the polo-like kinase 1 (Plk1) correlated to a poor prognosis in epidermoid carcinomas.

**Methods:** The molecular links between Plk1 and resistance to cisplatin/radiotherapy were investigated in patients and cell lines resistant to cisplatin and/or to radiotherapy. The therapeutic relevance of the Plk1 inhibitor onvansertib, alone or combined with cisplatin/radiotherapy, was evaluated on the proliferation/migration on HNSCC cell lines, in experimental HNSCC in mice, in a zebrafish metastasis model and on patient-derived 3D tumor sections.

**Results:** Plk1 expression correlated to a bad prognosis in HNSCC and increased after relapse on cisplatin/radiotherapy. Onvansertib induced mitotic arrest, chromosomic abnormalities and polyploidy leading to apoptosis of sensitive and resistant HNSCC cells at nanomolar concentrations without any effects on normal cells. Onvansertib inhibited the growth of experimental HNSCC in mice and metastatic dissemination in zebrafishes. Moreover, onvansertib combined to cisplatin and/or radiotherapy resulted in a synergic induction of tumor cell death. The efficacy of onvansertib alone and in combination with reference treatments was confirmed on 3D viable sections of HNSCC surgical specimens.

**Conclusions:** Targeting Plk1 by onvansertib represents a new strategy for HNSCC patients at the diagnosis in combination with reference treatments, or alone as a second line treatment for HNCSCC patients experiencing relapses.

## Introduction

Head and neck squamous cell carcinomas (HNSCC) represent the fifth most common cancer worldwide and cause high mortality every year due to the failure of reference treatments by surgery, cisplatin and radiotherapy. Currently, 50% of patients locally relapse and succumb to the disease because of resistance to chemotherapy and radiotherapy 1.

The principal risk factors of the pathology are tobacco, alcohol 2, 3 and human papillomavirus (HPV) infection 4.

HNSCC are characterized by a high proliferation rate and tumor cells that undergo resistance to standard therapies continue to proliferate uncontrollably. One of most important regulators of the cell cycle is the Polo like kinase 1 (Plk1). Plk1 is overexpressed in HNSCC and its overexpression correlates to a bad prognosis 5-7.

Polo was first identified in Drosophila melanogaster 8. The family of polo-like serine/threonine kinases is conserved in nearly all organisms and includes 5 members: Plk1, Plk2, Plk3, Plk4 and Plk5 9. Plk1 comprises a C-terminal polo-box domain which interacts with its substrates, and a N-terminal kinase catalytic domain to phosphorylate its substrates. Plk1 activates 622 substrates during the different steps of the cell cycle 10. It controls centromere maturation, mitosis entry, chromosome segregation and cytokinesis 11. It plays a key role in the DNA damage checkpoint 12 and it is an essential actor of cell proliferation and maintenance of genomic stability. Plk1 is activated by Aurora A and Bora 13 to initiate mitosis' entry. This major role in cell proliferation appears relevant to stop tumor progression by specific inhibitors of Plk1.

The depletion of Plk1 in cancer cells resulted in cell cycle arrest, polyploidy, and apoptosis 14, 15. Onvansertib, a Plk1-specific ATP competitive inhibitor, blocks the phosphorylation of Plk1 substrates 16. *In vivo*, onvansertib reduces osteosarcoma 17 and ovarian carcinoma growth 18 and it increases median survival of acute myeloid leukemia (AML) patients 19. Onvansertib is currently tested in solid metastatic cancers. Three phase II clinical trials are ongoing in AML (NCT03303339) and in metastatic prostate (NCT0341403) and colorectal (NCT03829410) cancers.

In this study, we showed that onvansertib is more efficient on HNSCC cell lines as compared to cell lines representative of other solid or hematologic cancers. We generated for the first time, HNSCC cell lines resistant to both cisplatin and radiotherapy. Inhibition of Plk1 by onvansertib induced mitotic catastrophe and the death of cisplatin/radiotherapy-sensitive and -resistant HNSCC cells. Onvansertib strongly inhibited the growth of experimental cisplatin/radiotherapy-sensitive and -resistant HNSCC tumors in mice and the metastatic dissemination of resistant HNSCC cells in zebrafishes. Moreover, we clearly showed that onvansertib combining with cisplatin and radiotherapy exerts a synergistic inhibition on cell proliferation *in vitro* but also on viable sections of surgical resections of HNSCC. These experiments, in strong collaboration with oncologists, pathologists and surgeons, showed that onvansertib represents a relevant drug for the treatment of HNSCC.

## Methods

### Cell lines

CAL33 (named 33) and CAL27 (named 27), two human head and neck cancer cell from the Centre Antoine Lacassagne have been used to establish cell lines resistant to cisplatin (CAL27 cis-R and CAL33 cis-R), to radiotherapy (CAL33 rad-R) or to both treatments (CAL33 RR and CAL27 RR).

CAL27 cis-R and CAL33 cis-R were obtained by cultivating the cells with increased concentrations of cisplatin until 10 µM. CAL33 rad-R cells were obtained after 25 rounds of 8 Gy irradiation. CAL33 RR and CAL27 RR were obtained from CAL33 cis-R and CAL27 cis-R and after 25 rounds of 8 Gy irradiation.

Human fibroblast (FHN) and keratinocytes, non-cancer cells, were obtained from ATCC. Cancer cell lines BT549, Detroit, 786, Mel202, DU145 and MDA-MB-231 were obtained from ATCC. NB4, U937, U266 and 8226 were kindly provided by Dr Patrick Auberger (C3M, Nice).

Cells were cultured in DMEM high glucose, GlutaMAXTM Supplement, pyruvate - (Thermo Fisher Scientific) and supplemented with 7% FCS and 0.1% penicillin streptomycin (10 000 μg/mL, Gibco® Life Technologies).

Keratinocytes (given by Dr Magnaldo Thierry) were obtained as waste tissues following plastic mammary surgery of a 40 years-old female patient after informed consent according to approval of the local ethical comity. After trypsin/EDTA dissociation, primary keratinocytes were cultured on a feeder layer of lethally irradiated 3T3-J2 Swiss mouse fibroblasts (3T3-J2) in cFAD medium as described by Rheinwald and Green 20. When reaching about 60% confluency, mouse cells feeder layer was removed by 5 min incubation in 0.02% EDTA. Then, 48 h before harvesting, sub-confluent keratinocytes were washed twice in PBS and then solubilized in cell lysis buffer, followed by sonication treatment.

### Immunoblotting

Cells were lysed in SDS 7.5%; glycerol 30%; Tris 0.3 M pH 6.8 lysis buffer.

30 µg of proteins were separated on 10% SDS-polyacrylamide gels and transferred on PVDF membranes. The following primary antibodies were used: Plk1 (Abcam, ab17056, mouse) and ARD1 (home-made antibody, rabbit), Histone H3 (Cell signaling, #9715), phospho-Histone H3 (Cell signaling, #53348), NPM (Cell signaling, #3542), phospho-NPM (Cell signaling, #3541), PARP (Cell signaling, #9532), HSP90 (Cell signaling, #4877), HSP60 (Cell signaling, #12165), GAPDH (Cell signaling, #2118), phospho-TCTP (Cell signaling, #5251), phospho-Cdc25C (Cell signaling, #9529).

### RNA interference

Plk1 siRNA was purchased from Ambion (Plk1, Silencer Select siRNA, Standard, Cat#: 4390824). Control siRNA was purchased from Sigma (Universal, Negative Silencer #1). 150 000 cells were plated and transfected with Plk1 siRNA or Control siRNA (50 nM/ well for 6 well culture plates) using the 500 μL of opti-MEM medium (Gebco) in presence of 5 μL of Lipofectamine RNAiMAX (Invitrogen) and 2 mL of DMEM medium without penicillin streptomycin. After 48 h, 96 h and 120 h post- transfection, cells were analyzed.

### Flow cytometry

50 000 cells of each line were cultured in a 6-wells plates with onvansertib (Selleckchem, NMS-P937, NMS1296937) and/or cisplatin for 48 h.

Apoptotic and dead cells were stained with Annexin V-APC (Biolegend, 8 μg/ml) and propidium iodide (PI, Biolegend, 0.5 mg/ml) for 15 min at 4 °C and analyzed with FACSCalibur cytometer (BD Biosciences).

To analyze cell cycle and polyploidy, 200 000 cells were cultured with onvansertib or cisplatin for 24 h and conserved in ethanol 70% at -20 °C. They were incubated in PBS containing 3 μg/ml RNase A and 40 μg/ml PI for 30 min at 4 °C and analyzed with FACSCalibur cytometer (BD Biosciences).

### XTT assays

5000 cells exposed to different concentrations of onvansertib and/or cisplatin, were cultured in 96-wells plates for 48 h. XTT tests (Cell Proliferation kit II, Sigma-Aldrich®) allowed to measure cell metabolism at 490 nm (Promega GloMax®-Microplate).

### Colony formation assays

3000 cells were treated with onvansertib and/or cisplatin and/or irradiated at 2 and 4 Gy. After 10 days, clones were colored with GIEMSA (Sigma-Aldrich®).

### Migration assays

50 000 cells were cultured in DMEM 0% FCS and seeded in Boyden chambers. After 24 h, Boyden chambers were washed with PBS. Migrative cells were fixed with paraformaldehyde 3% and colored with crystal violet.

### Invasion assays

2000 cells were plated in 48 multi-wells plates covered with agarose 0.8%. After two days, spheroids were mixed with 200 µl matrigel (Corning® Matrigel® Matrix), 100 µl collagen I (8 mg/ml; corning), 80 µl HEPES 1M and DMEM 7% FCS in 12 multi-wells plates. 1 h after DMEM 7% FCS was added with or without treatment. Pictures were recorded every day.

### Staining by hematoxylin eosin safran (HES)

10 000 cells were seeded in control condition or in the presence of onvansertib. The staining was performed by the pathology department of the University Hospital of Nice (Dr Ambrosetti).

### Analyses by RT-qPCR

RNA from cells were purified with the RNeasy Mini Kit (Quiagen). The “QuantiTect Reverse Transcription Kit” (Quiagen) was used for cDNA obtention. The PCR program was executed on “Professional Basic Thermocycler” (Biometra). SYBR master mix plus (Eurogentec) was used for qPCR. The mRNA level was normalized to 36B4 mRNA.

### Mice models

10^6^ CAL33 and CAL33RR cells were injected subcutaneously into the flanks of 5-week-old nude (nu/nu) female mice (Janvier, France). Tumor volume was determined with a caliper (v = L*l^2^*0.5). When the tumor reached 80 mm^3^, mice were treated five times a week for 4 weeks by gavage with onvansertib (60 mg/kg), and once a week for 4 weeks with cisplatin (4 mg/kg) administered intraperitoneally. This study was carried out in strict accordance with the recommendations of the Guide for the Care and Use of Laboratory Animals. Our experiments were approved by the ''Comité national institutionnel d'éthique pour l'animal de laboratoire (CIEPAL)'' (reference: 2018102510495275 - PEA 535).

### Zebrafish models

All animal experiments were approved by the Northern Stockholm Experimental Animal Ethical Committee. Zebrafish embryos were raised at 28°C under standard experimental conditions. Zebrafish embryos at the age of 24-hpf were incubated in water containing 0.2 mmol/L 1-phenyl-2-thio-urea (PTU, Sigma). At 48-hpf, zebrafish embryos were dechorionated with a pair of sharp-tip forceps and anesthetized with 0.04 mg/mL of tricaine (MS-222, Sigma). Anesthetized embryos were subjected for microinjection. CAL33 RR tumor cells were labeled *in vitro* with a Vybrant DiD cell-labeling solution (LifeTechnologies). Tumor cells were resuspended in PBS and approximatively 5 nL of the cell solution were injected into the perivitelline space (PVS) of each embryo by an Eppendorf microinjector (FemtoJet 5247). Non-filamentous borosilicate glass capillaries needles were used for injection and the injected zebrafish embryos were immediately transferred into PTU aquarium water. Fish are immediately treated with onvansertib (50 nM). For Figure 5E-F, 24 h after injection, only zebrafish with metastasis were chosen and treated with onvansertib (50 nM). Zebrafish embryos were monitored 72 h for investigating tumor metastasis using a fluorescent microscope (Nikon Eclipse 90).

### Patients

All patients gave written consent for the use of tumor samples for research. This study was conducted in accordance with the Declaration of Helsinki.

#### TCGA cohort: HNSCC patients - Plk1 mRNA analysis

Normalised RNA sequencing (RNA-Seq) data produced by The Cancer Genome Atlas (TCGA) were downloaded from cBioportal (www.cbioportal.org, TCGA Provisional; RNA-Seq V2). Data were available for 530 HNSCC tumor samples TCGA subjected to mRNA expression profiling.

DFS and OS were calculated from patient subgroups with Plk1 mRNA levels (z-score) that were less or greater than the third quartile value.

#### French cohort: HNSCC patients from Centre Antoine Lacassagne

Centre Antoine Lacassagne pathology department provided us with paraffin embedded samples of HNSCC at diagnosis and relapse. These samples have been deparaffinized and RNA has been extracted with RNeasy FFPE Kit (Quiagen 73504). qPCR has been performed to measure Plk1 expression.

### Treatment of primary HNSCC viable 3D sections - HNSCC patients from Centre Antoine Lacassagne

HNSCC tumors obtained just after surgery were provided through a collaboration with the Centre Antoine Lacassagne (Pr Bozec). Viable tumor sections (250 µm, tumor samples were obtained with a vibratome HM650V (Thermo Scientific) and the presence of tumor cells was confirmed by a pathologist (Dr Boyer). They were seeded in Airway Epithelial Cell Growth Medium (PromoCell) supplemented with Normocure^TM^ (Invivogen) and treated with a range of onvansertib, cisplatin concentrations or radiotherapy, alone or in combination. The ATP concentration (ATP Bioluminescence Assay Kit CLS II, Roche) was measured in the lyzed tumor sections and represented a read-out of their viability. Tumor sections were then paraffin-embedded and analysed using HES for the quantification of necrotic areas or KI-67 staining to quantify cell proliferation. Tumor sections were then deparaffined and treated by TUNEL immunofluorescence (*In situ* Cell Death Detection Kit, Fluorescein, Roche) to evaluate the amounts of apoptotic cells.

### Immunohistochemistry

#### Xenograft model

Experimental HNSCC were embedded in paraffin for immunostaining. Tumor sections were incubated with CD31 (clone MEC 13.3, BD Pharmingen, diluted at 1:500), Ki67 (clone MIB1, DAKO, Ready to use) or αSMA (clone 1A4, DAKO, Ready to use).

### Statistical analysis

#### For *in vitro* and *in vivo*

Statistical analyses were performed with Prism 8 software. Results are expressed as the mean +/- the standard error (SEM). Significance was determined by Student's t-test. ANOVA with Bonferroni post hoc test was used for multiple comparisons.

#### For patients

The Student's t-test was used to compare continuous variables and chi-square test, or Fisher's exact test (when the conditions for use of the χ^2^-test were not fulfilled), were used for categorical variables. DFS was defined as the time from surgery to the appearance of metastasis. PFS was defined as the time between surgery and progression, or death from any cause, censoring live patients and progression free at the last follow-up. OS was defined as the time between surgery and the date of death from any cause, censoring those alive at the last follow-up. The Kaplan-Meier method was used to produce survival curves and analyses of censored data were performed using Cox models.

#### For clonogenicity tests

Analyses of synergic effect has been done by the statistic department of the Centre Antoine Lacassagne. Potentialisation effect (synergy or antagonism) were evaluated by using interaction test for linear regression model. An interaction with p < 0.05 will be considered as significant (Figure S5E).

## Results

### Plk1 is strongly expressed in HNSCC, is associated with a poor prognosis and its expression increased at relapse on cisplatin/radiotherapy first-line treatment

**Figure 1:**
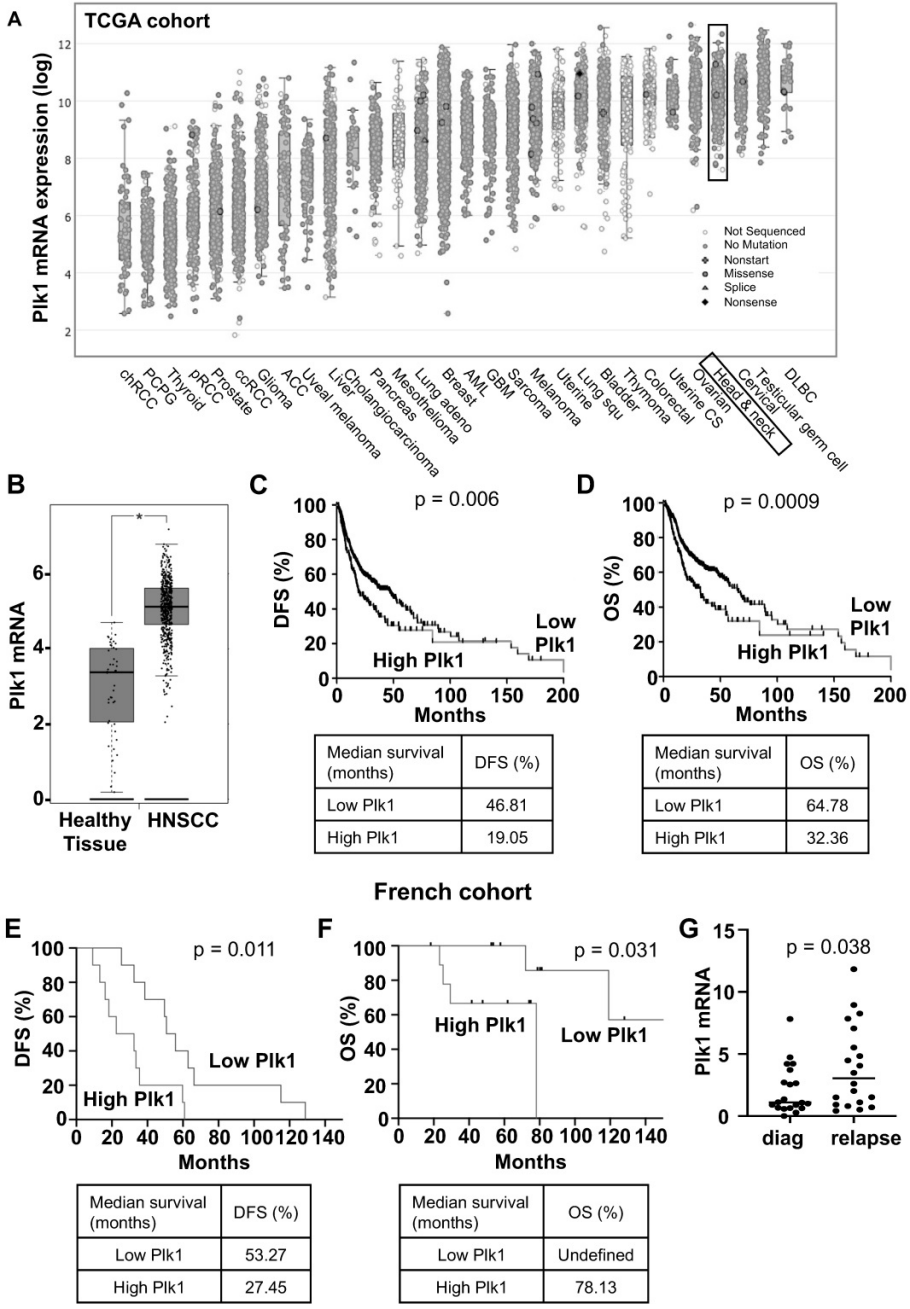
** Plk1 is overexpressed in HNSCC patients and is associated with a poor prognosis.** The tumors of HNSCC patients were analyzed for Plk1 mRNA levels (z-score). These results are in whole based upon data generated by the TCGA Research Network. **(A)** Comparison of Plk1 mRNA levels in several cancer. **(B)** Comparison of Plk1 mRNA levels between healthy (n = 44) and HNSCC tissues (n = 519). **(C-D)** The levels of Plk1 mRNA in tumors of HNSCC patients correlated with DFS (**C**) and OS (**D**). **(E-F)** The levels of Plk1 mRNA in patients' tumors from Centre Antoine Lacassagne correlated with DFS (**E**) and OS (**F**). DFS and OS were calculated from patient subgroups with mRNA levels that were less or greater than the third quartile value. Statistical significance (p value) is indicated. **(G)** Plk1 mRNA measured by qPCR in tumors from Centre Antoine Lacassagne's patients at diagnosis and after relapse to usual treatments (n = 20).

Plk1 mRNA levels have been determined in solid cancers using the TCGA database. Plk1 mRNA levels are high in kidney, lung, breast, ovarian, colon cancers, melanoma and particularly in HNSCC (Figure 1A). Plk1 mRNA levels were higher in HNSCC as compared to healthy tissue (*p* < 0.0001, Figure 1B). They were also increased in node-positive tumors (N1, aggressive and invasive tumor, p = 0.035, Figure S1A). However, Plk1 mRNA levels did not depend on the HPV status (Figure S1B). In HNSCC from the TCGA cohort, high levels of Plk1 mRNA correlated with shorter disease-free survival (DFS, 19.05 months vs 46.81 months, *p* = 0.006, Figure 1C) and shorter overall survival (OS, 32.36 months vs 64.78, *p* = 0.0009, Figure 1D). Equivalently, in HNSCC from the Centre Antoine Lacassagne (French cohort, Table S1), high levels of Plk1 mRNA correlated with shorter DFS (27.45 months vs 53.27 months, *p* = 0.011, Figure 1E) and shorter OS (78.13 months vs undefined, *p* = 0.031, Figure 1F). Plk1 is overexpressed in HNSCC that relapse after first-line treatment with cisplatin and radiotherapy (*p* = 0.038, Figure 1G). These observations suggested that Plk1 represents a marker of poor prognosis and confirmed previous results 7. Our results suggested that Plk1 is also a marker of relapse/resistance in HNSCC and could represent a relevant therapeutic target for these cancers at the diagnosis as previously suggested 21, 22 but also at relapse on reference treatments by chemo/radiotherapy.

### Cisplatin and/or radiotherapy-resistant cells are more aggressive

**Figure 2:**
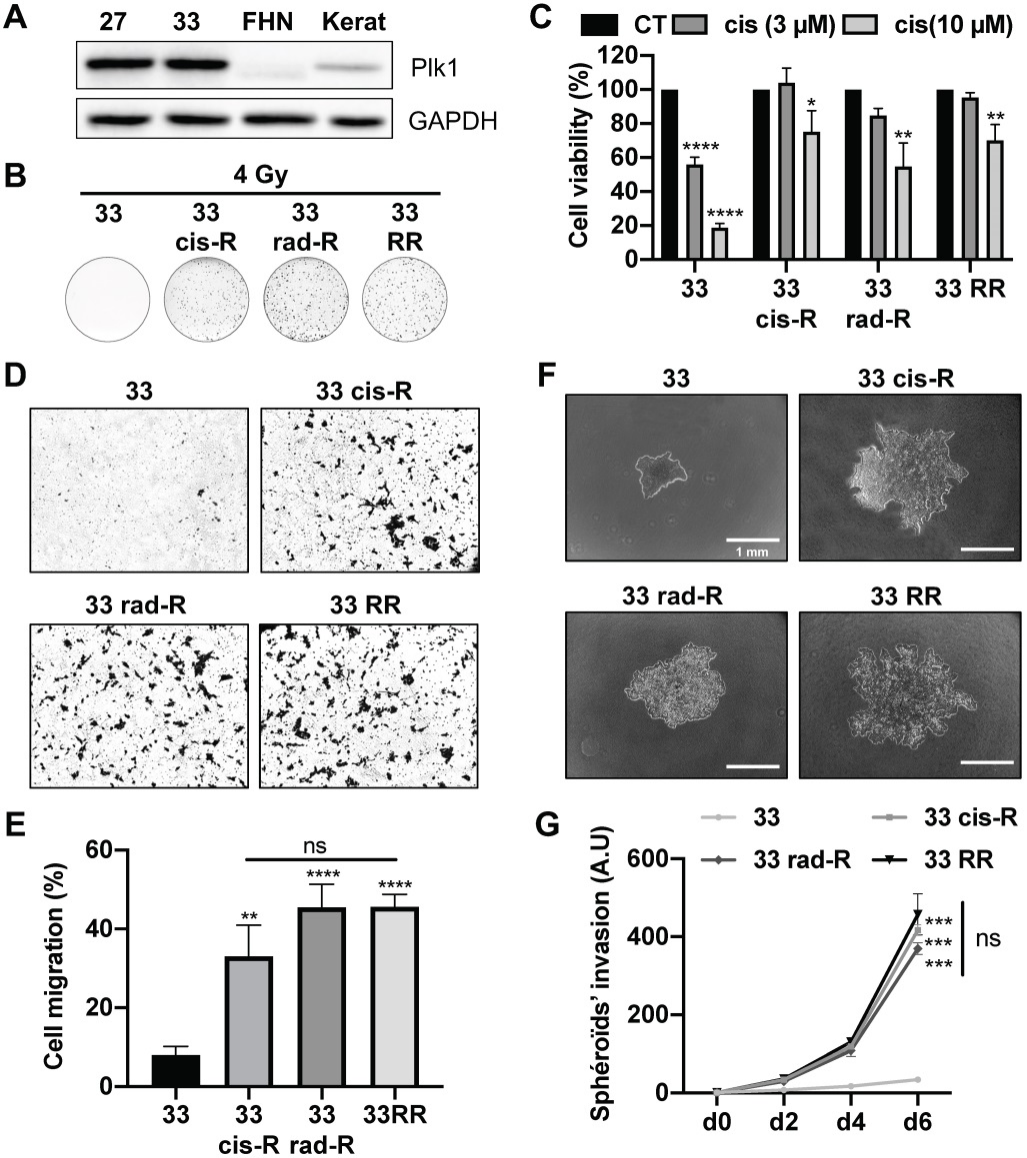
** Characterization of CAL33 sensitive and resistant cell lines.** (**A**) Plk1 immunoblotting on keratinocytes (kerat), human fibroblasts (FHN), CAL27 and CAL33 cells. GAPDH served as a loading control. (**B**) Clonogenicity assay of CAL33, CAL33 cis-R, CAL33 rad-R and CAL33 RR after 4 Gy of radiation (n = 2). (**C**) Cell viability of CAL33, CAL33 cis-R, CAL33 rad-R and CAL33 RR after 48 h of cisplatin treatment (3 and 10 µM) (n = 3). (**D-E**) Cell migration using Boyden's chamber assay on CAL27 and CAL33 sensitive and resistant cell lines. Results are expressed as the percentage of the control (n = 3). (**F-G**) Spheroids' invasion using 3D culture cell assays on sensitive and resistance CAL33 and CAL27 cells (n = 2). Results are expressed as arbitrary units (day 0 used as the control). Statistics were performed using ANOVA test: * *p* < 0.05, ** *p* < 0.01, *** *p* < 0.001, **** *p* < 0.0001.

CAL33 and CAL27 cells resistant to cisplatin, radiotherapy or both have been generated. Curiously, no CAL27 cells only resistant to radiotherapy could be obtained, suggesting that resistance to cisplatin favors resistance to radiotherapy. CAL33 and CAL27 cells overexpressed Plk1 as compared to human fibroblasts (FHN) and keratinocytes, two healthy cell types (Figure 2A). Plk1 was expressed to a comparable extent in sensitive and resistant CAL27 and CAL33 cells that already overexpress Plk1 (Figure S2A).

A clonogenicity test performed on CAL27 and CAL33 sensitive and resistant cells after a 4 Gy irradiation confirmed resistance to radiotherapy (Figure 2B; Figure S2B). CAL33 Cis-R and CAL27 Cis-R cells formed clones after irradiation, suggesting cross-resistance mechanisms.

CAL33 and CAL27 cell viability was strongly decreased after exposure to moderate and high concentrations of cisplatin (3-10 μM) which was not the case for all resistant cells (Figure 2C; Figure S2C). CAL33 Rad-R were resistant to cisplatin suggesting a “cross resistance”.

All resistant CAL33 cells acquired migration abilities but the ability to migrate was more important for sensitive as compared to resistant CAL27 cells (Figure 2D-E; Figure S2D and S2E). This result suggests that CAL27 cells are intrinsically more aggressive as compared to CAL33 cells. In 3D cell culture conditions, CAL33 and CAL27 resistant cells were more invasive as compared to sensitive cells (Figure 2F-G; Figure S2F-G). These observations strongly suggest that cisplatin and/or radiation resistant cells acquired a more aggressive phenotype.

### Onvansertib, a Plk1 inhibitor, is efficient on sensitive and resistant HNSCC cells and enhances cell death by mitotic catastrophe and apoptosis

The efficacy of onvansertib was studied on several types of cancer cells including HNSCC. The translationally controlled tumor protein (TCTP) and the dual specificity phosphatase Cdc25 are both substrates of Plk1. The inhibition of Plk1 by onvansertib decreases the phosphorylation (specific phosphorylation site by Plk1) of these two substrates. This result favors the specificity of action of onvansertib (Figure S3A).

The lowest IC50 was obtained for HNSCC cells as compared to the other cancer cell lines. Onvansertib showed low toxicity on healthy cells even at high concentrations (250 nM, Table S2). The low toxicity and the efficacy at low concentrations suggest the relevance of onvansertib for the treatment of HNSCC. The efficacy of onvansertib assessed by viability tests was confirmed by clonogenicity tests with increasing concentrations of onvansertib, cisplatin and irradiation doses (Figure S3B-C).

**Figure 3:**
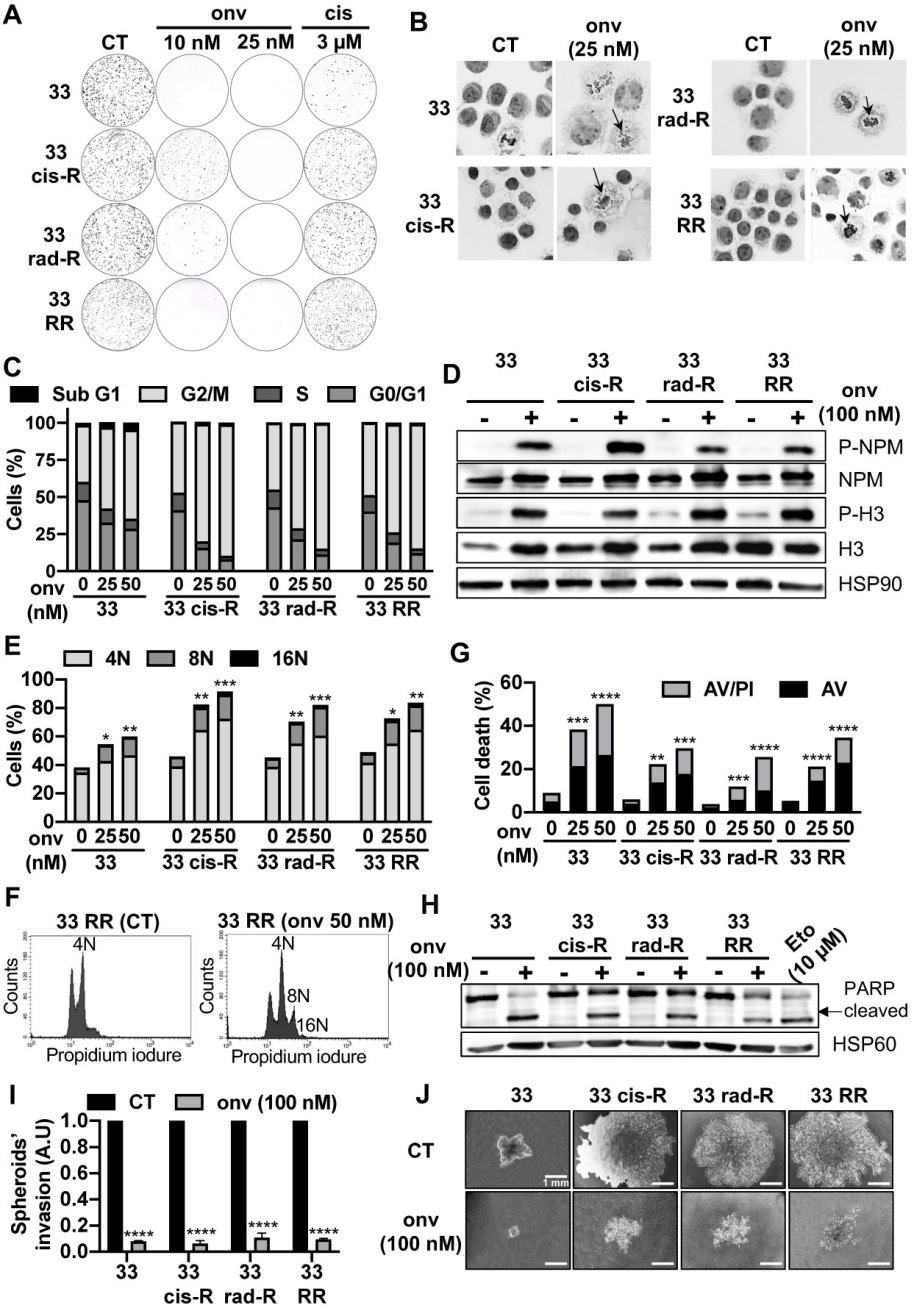
** Onvansertib effects on sensitive and resistant CAL33 cell lines. (A)** Clonogenicity assay of sensitive and resistant CAL33 cell lines after onvansertib (10 nM and 25 nM) or cisplatin (3 μM) treatment (n = 2). **(B)** Hematoxylin eosin saffron staining of sensitive and resistant CAL33 cell lines treated with onvansertib (25 nM) for 24 h. Abnormal mitosis was indicated with an arrow. **(C-D)** Cells were treated with onvansertib (25 nM, 50 nM or 100 nM) for 24 h. Cell cycle was measured by flow cytometry (n = 3) (**C**), G2/M arrest was evaluated by phospho-NPM and, phospho-H3 immunoblotting. HSP90 served as a loading control (n = 2) (**D**). **(E-F)** Polyploidy (number of nuclei 8N and 16N) was assessed by flow cytometry (n = 3) (**E**). Representation of polyploidy in CAL33 RR (**F**). **(G)** Cells were treated with onvansertib (25 nM and 50 nM) for 48 h. Cell death was evaluated by flow cytometry. Cells were stained with propidium iodure (PI) and Annexin V (AV). Histograms show AV^+^/PI^-^ cells (early apoptosis) and AV^+^/PI^+^ cells (late-apoptosis or another cell death) (n = 3). **(H)** Immunostaining of PARP expression on CAL33 cell lines after exposure to onvansertib (100 nM) for 24 h. HSP60 served as loading control (n = 2). **(I-J)** Invasion of sensitive and resistant CAL33 cells using 3D cell culture assay (spheroids) after 6 days of onvansertib treatment (100 nM) grown in (**I**). Results are represented as arbitrary units (the no-treatment condition was used as a control) (n = 2) (**J**). Statistics were analyzed using ANOVA tests: * *p* < 0.05, ** *p* < 0.01, *** *p* < 0.001, **** *p* < 0.0001.

Onvansertib inhibited the formation of naïve CAL33 and CAL27 cells from 10 nM (Figure 3A; Figure S4A). Some clones persisted at 3 µM of cisplatin. Onvansertib 25 nM inhibited the formation of clones with all resistant CAL33 and CAL27 cells (Figure 3A; Figure S4A) while 3 µM of cisplatin did not. This experiment suggested the relevance of using onvansertib at relapse on cisplatin/radiotherapy.

Since Plk1 plays a central role in mitosis' entry 23 , the impact of onvansertib on the cell cycle was evaluated. Onvansertib induced a misalignment of the chromosomes and therefore a mitotic defect in CAL33 cells (Figure 3B). Onvansertib treatment induced the accumulation of all sensitive and resistant CAL33 and CAL27 cells in the G2/M phase in a dose dependent manner (Figure 3C; Figure S4B). The phosphorylation on serine 10 of histone 3 (p-H3) and phosphorylation on threonine 199 of nucleophosmin (p-NPM) are hallmarks of condensation of chromosomes 24, duplication of centrosomes and mitosis 25, 26. Onvansertib increased the phosphorylation of histone H3 and NPM and clearly induced mitotic arrest of CAL33 and CAL27 cells (Figure 3D; Figure S4C). Basal polyploidy was observed in control condition, however onvansertib enhanced polyploidy with an increased number of nuclei (accumulation of nuclei, from 8 to 16N, Figure 3E and Figure S4D) as it was shown for CAL27 RR and CAL33 RR (Figure 3F; Figure S4E). These observations are characteristics of the mitotic catastrophe leading to cell death.

Onvansertib induced the apoptosis of sensitive and resistant CAL33 and CAL27 cells in a dose dependent manner (annexinV/propidium iodure positive cells, Figure 3G; Figure S4F; PARP cleavage, Figure 3H; Figure S4G).

We confirmed that decreased expression of Plk1 expression by Plk1 siRNA induced apoptosis cell death like onvansertib (Figure S4H).

Onvansertib also inhibited the growth of spheroids and invasive properties of sensitive and resistant CAL33 and CAL27 cells (Figure 3I-J; Figure S4I). Therefore, by targeting Plk1, onvansertib inhibits several hallmarks of cancer aggressiveness *in vitro*.

### Onvansertib inhibits the growth of experimental HNSCC in nude mice

**Figure 4:**
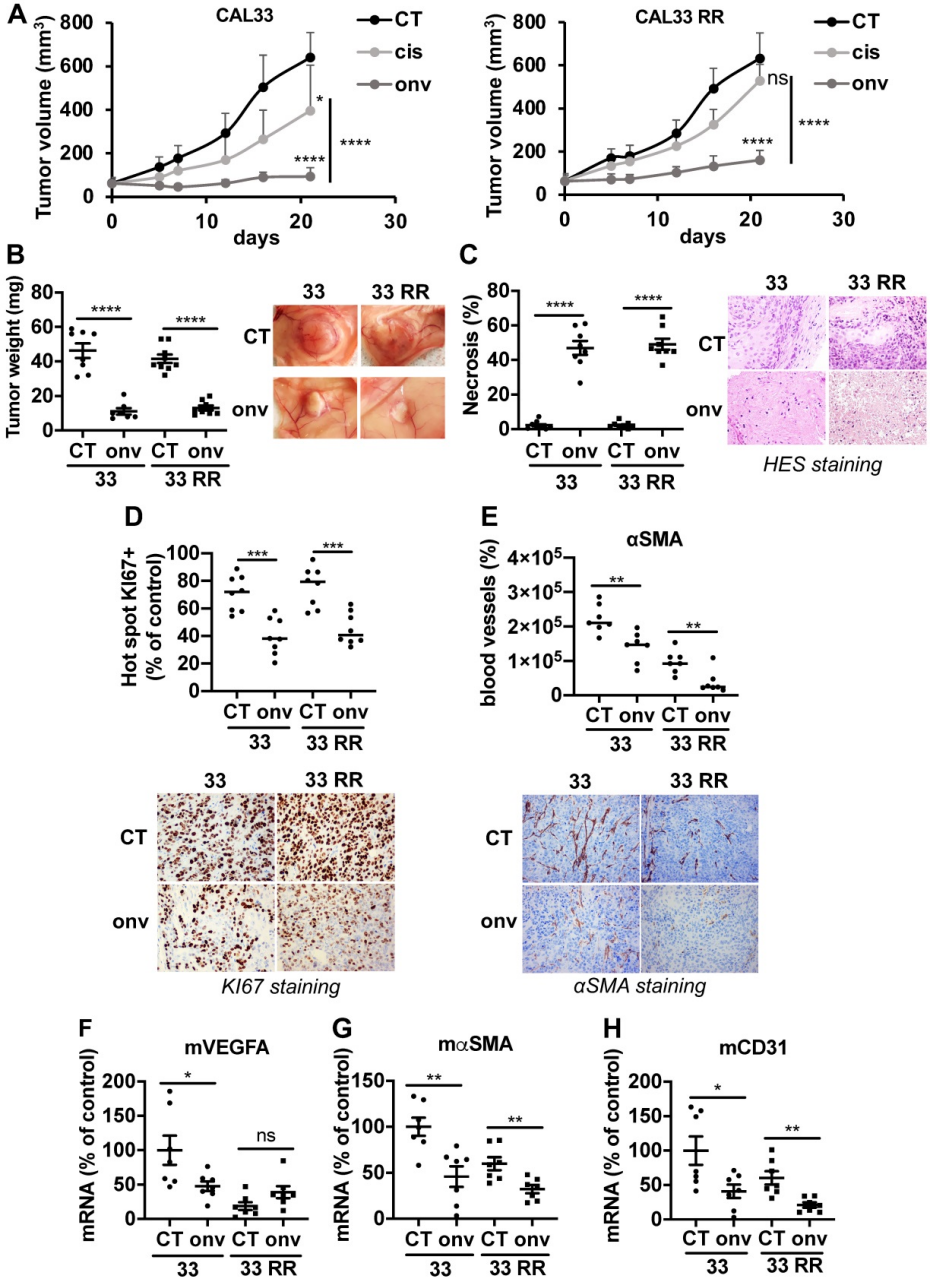
** Onvansertib inhibits the growth of experimental tumors in mice.** 10^6^ CAL33 or CAL33 RR cells were subcutaneously injected in the flank of nude mice. When the tumors reached 100 mm^3^, mice were treated with onvansertib (60 mg/Kg) or with cisplatin (4 mg/Kg). (**A**) The tumor volume was measured twice a week. (**B**) At the end of the experiment, the weight of tumors has been evaluated and representative pictures of tumors treated or not with onvansertib are shown. (**C**) Necrosis was quantified in control and onvansertib-treated tumors (HES staining). **(D)** Pictures and quantification of proliferative cells measured by KI-67 staining. **(E)** Pictures of blood vessels and quantification evaluated by IHC of αSMA. **(F-H)** Murine VEGFA (mVEGFA, F), αSMA (mαSMA, **G**) and CD31 (mCD31, **H**) mRNA levels in tumors were determined by qPCR. Results are expressed as percent of control. Statistics were performed using an unpaired Student's t test: * *p* < 0.05, ** *p* < 0.001, **** *p* < 0.0001.

Experimental tumors were generated by xenografting CAL33 and CAL33 RR in nude mice. Onvansertib and cisplatin efficacy was compared when tumors reached 100 mm^3^. Onvansertib strongly reduced the growth of tumors generated with sensitive (CAL33) cells while cisplatin was less efficient (Figure 4A). Cisplatin did not affect the growth of tumors generated with resistant (CAL33 RR) cells confirming the inefficacy observed *in vitro*. However, onvansertib inhibited the growth of tumors generated with CAL33 RR as efficiently as those generated with sensitive cells (Figure 4A). The onvansertib-treated tumors were smaller (Figure 4B) and necrotic (Figure 4C). The number of proliferating cells assessed by KI-67 (Figure 4D) and the blood vessels density determined by αSMA staining by IHC (Figure 4E) or VEGFA, αSMA and CD31 mRNA levels (Figure 4F-H) were reduced in onvansertib-treated tumors. These results strongly suggest that onvansertib efficacy not only relies on the blockade of tumor cell proliferation but also on the inhibition of angiogenesis.

### Onvansertib inhibits metastatic spreading and the growth of metastases in the zebrafish

**Figure 5:**
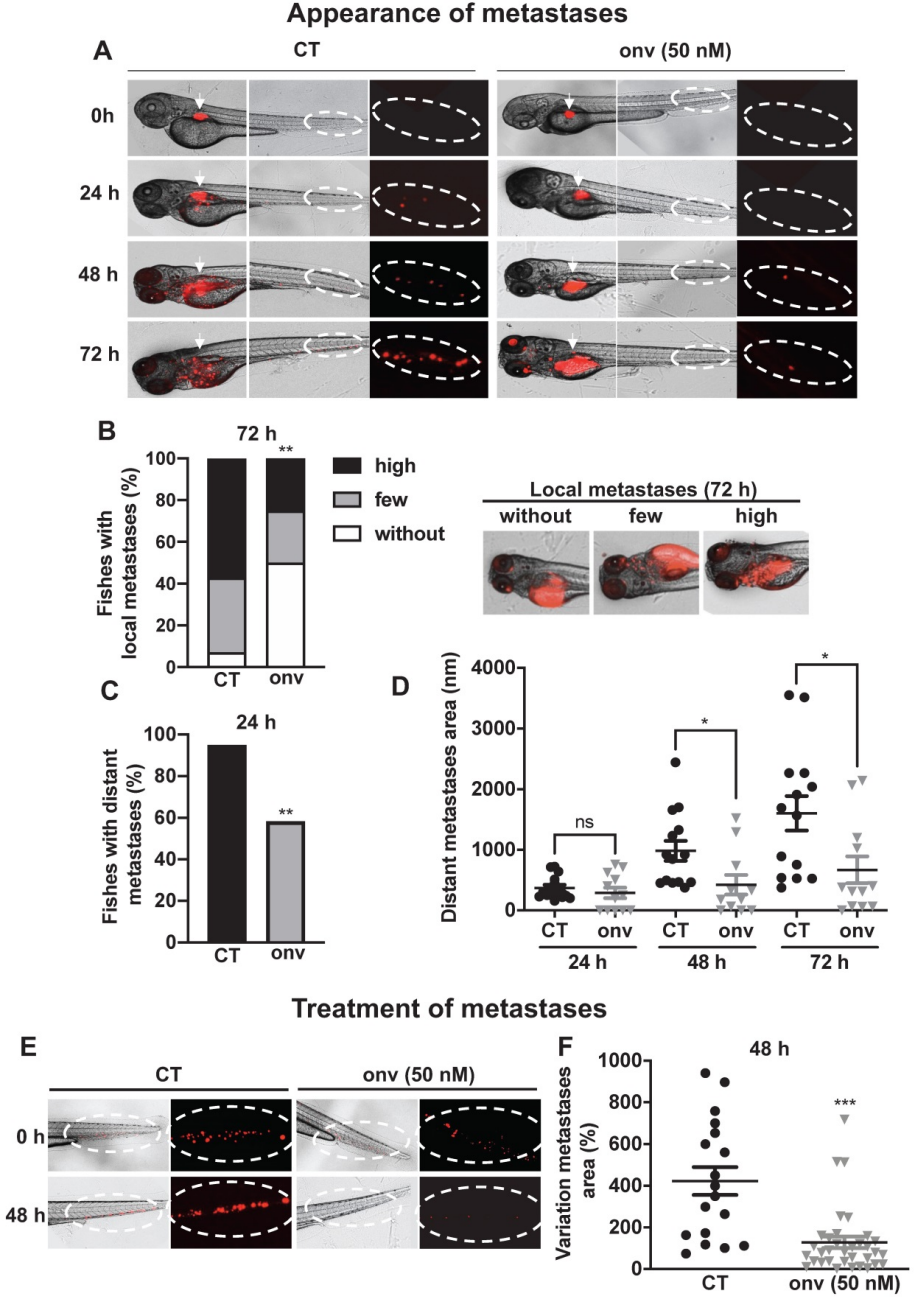
** Onvansertib inhibits the formation and growth of CAL33 RR metastases in zebrafish**. (**A**-**D**) Zebrafish embryos (n = 28) were injected with CAL33 RR (labelled in red) into the perivitelline space and immediately treated with onvansertib (50 nM) for 72 h. Representative images of zebrafishes are shown at 24 h, 48 h and 72 h (**A**). Quantification and representative images of no, few and high local metastases in the head after 72 h of treatment (**B**). Percentage of zebrafishes with tumor cells in the tail (distant metastasis) at 24 h were quantified (**C**). Area of distant metastases at 72 h were quantified (**D**). (**E**-**F**) Zebrafish embryos (n = 48) were injected with CAL33 RR (labelled in red) into the perivitelline space. 24 h later, zebrafish embryos with metastases were treated with onvansertib (50 nM) for 48 h. Representative image of zebrafish are shown (**E**) and variations of the metastases area before and after treatment were quantified (**F**). Statistics were performed using an unpaired Student's *t test*: ** *p <* 0.01, *** *p <* 0.001.

Zebrafishes were used as a relevant model of metastatic dissemination of tumor cells from the site of injection to the tail 27. Onvansertib inhibited the local invasion after 72 h of treatment (Figure 5A-B) and the formation of distant metastases from 24 h (Figure 5A-C). From 48 h, onvansertib decreased the size of distant metastases that settled in the tail (Figure 5D) compared to the control group. Onvansertib reduced the size (more than 60% reduction) and the number of already existing distant metastases (Figure 5E-F). These results suggest that onvansertib is as efficient on primary tumors as on metastases.

### Synergic effect of onvansertib with cisplatin and radiotherapy

To determine a potential synergy, we chose concentrations/doses of each treatment that do not affect clone formation (onvansertib: 5 nM, cisplatin: 1 μM, irradiation: 2 Gy, Figure S5). In all CAL27 and CAL33 cells, the decrease of clones was obtained with onvansertib 5 nM/2 Gy of irradiation (Figure S5A-B) and onvansertib 5 nM/cisplatin 1 μM (Figure S5C-D).

**Figure 6:**
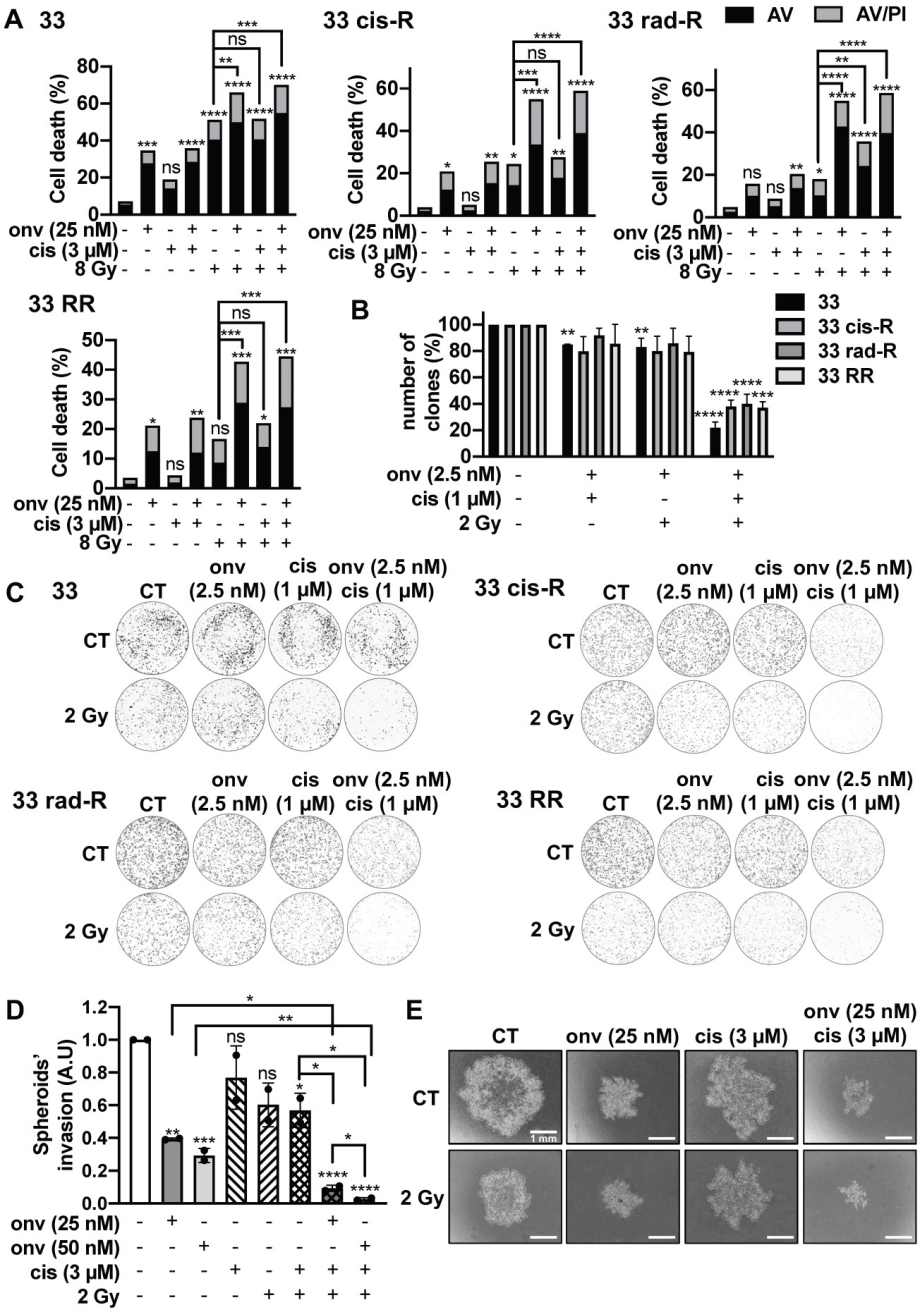
** Onvansertib effects in combination with reference treatments (cisplatin and radiotherapy) on sensitive and resistant CAL33 cell lines. (A)** Cells were treated 72 h with onvansertib (25 nM) in combination with cisplatin (3 µM) and radiotherapy (8 Gy). Cell death was evaluated by flow cytometry (n = 3). Cells were stained with Propidium Iodure (PI) and Annexin V (AV). Histograms show AV^+^/PI^-^ cells (early-apoptosis) and AV^+^/PI^+^ cells (late-apoptosis or another cell death). **(B-C)** Cells were treated with onvansertib (2.5 nM), cisplatin (1 µM) and radiotherapy (2 Gy). Quantification (**B**) and representative image of clonogenicity assay (C) (n = 3). **(D-E)** 3D cell culture assay (spheroid) was treated with the combination of the three treatments: onvansertib (25 nM and 50 nM), cisplatin (3 µM) and radiation (2 Gy) and evaluated after 6 days. Quantification (**D**) and representative image of invasion assay (**E**) (n = 2). Statistics were analyzed using ANOVA test: * *p* < 0.05, ** *p* < 0.01, *** *p* < 0.001, **** *p* < 0.0001 and unpaired Student's *t* test: * *p* < 0.05, ** *p* < 0.01, *** *p* < 0.001, **** *p* < 0.0001.

For a clinical perspective we then tested the triple combination. Sensitive and resistant CAL27 and CAL33 cells were irradiated at 8 Gy and exposed to onvansertib 25 nM and cisplatin 3 µM for 72 h (higher but suboptimal doses of each treatment because of a short exposure). Apoptotic cell death increased in sensitive and resistant cells (Figure 6A; Figure S6A). For colony formation assays, we used 2.5 nM onvansertib (Onvansertib 5 nM was lethal when used in a triple combination), cisplatin 1 µM and 2 Gy of irradiation. The number of clones was reduced as compared to the double combination experiments (Figure 6B-C; Figure S5).

The triple combination (onvansertib 25 nM, cisplatin 3 µM and 2 Gy) reduced the invasive properties of CAL33 RR and CAL27 RR grown in 3D (Figure 6D-E; Figure S6D-E).

These results suggest that onvansertib could be associated with low concentrations of cisplatin and low doses of irradiations to limit toxicity however inducing a robust therapeutic effect.

### Onvansertib alone or in combination with reference treatments induces the death of tumor cells in sections from surgical specimens of HNSCC

HNSCC were operated from six patients, and biopsies were analysed by a pathologist to choose viable parts of the tumors. Two samples correspond to primary HNSCC and three to local relapses after cisplatin and radiotherapy (Table S3).

**Figure 7:**
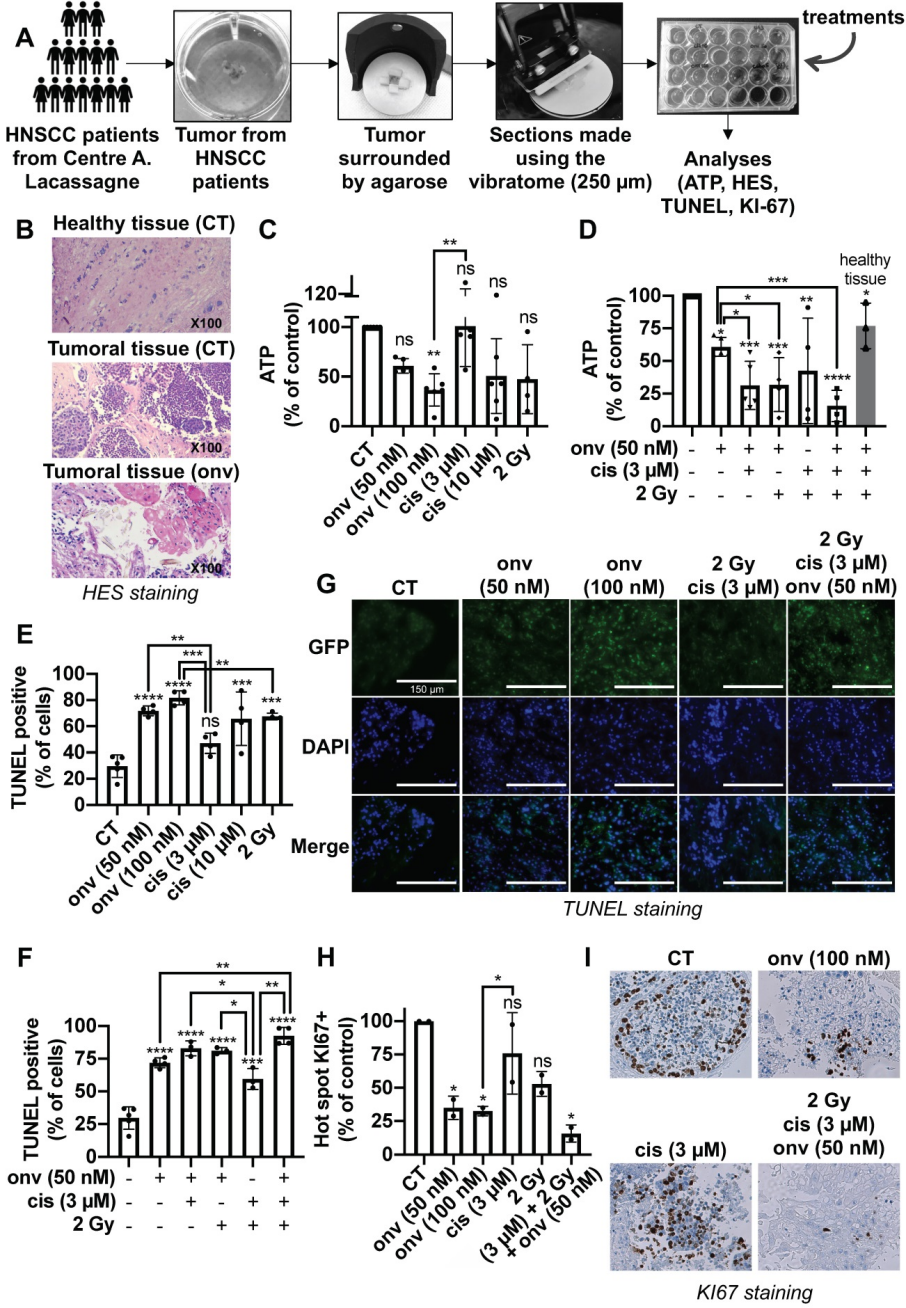
** Onvansertib on human tumor sections from HNSCC patients. (A)** Tumors from HNSCC patients surrounded by agarose and tumor sections were done using a vibratome. These sections were cultured in a specific medium and treated for 4 days with onvansertib or/and cisplatin or/and radiotherapy. Analyses were then performed (n = 6). **(B)** Necrosis of biopsies' sections assessed using HES (Hematoxylin Eosin Saffran) staining. **(C-I)** Biopsies' sections treated with onvansertib (50 nM and 100 nM), cisplatin (3 μM and 10 μM) or radiotherapy (2 Gy) (**C, E** and **H**), or treated with onvansertib alone or in combination with cisplatin and radiotherapy (**D, F** and **H**). **(C-D)** ATP quantification of biopsies' sections. **(E-G)** Quantification of cell death by apoptosis by TUNEL assays on biopsies' sections. **(H-I)** Pictures and quantification of cell proliferation evaluated by KI-67 staining. Statistics were performed using ANOVA test: * *p* < 0.05, ** *p* < 0.01, *** *p* < 0.001, **** *p* < 0.0001 and unpaired Student's t test: * *p* < 0.05, ** *p* < 0.01, *** *p* < 0.001, **** *p* < 0.0001.

250 microns sections of these different samples were obtained using a vibratome and contained all the cell populations of the tumor environment. They were treated with increasing concentrations of onvansertib and cisplatin for 4 days. ATP levels were then quantified as a read out of cell viability (illustration of the process in Figure 7A). HES staining detected the different architecture of healthy and tumor tissue. Onvansertib (100 nM) induced necrosis (Figure 7B). Onvansertib (100 nM) and irradiation (2 Gy) presented a high and equivalent efficacy as compared to cisplatin (3 μM). However, irradiation was more toxic on healthy tissue as compared to onvansertib (Figure S7). Moreover, we observed differences in the viability of sections treated with cisplatin or irradiation, suggesting an heterogenous response of the patients (Figure 7C). In combination with cisplatin (3 μM) and/or irradiation (2 Gy), onvansertib at 50 nM presented a higher efficacy as compared to the treatment alone (Figure 7D). This association showed a low toxicity on healthy tissues (Figure S7). The reference treatments induced heterogeneous efficacy suggesting that half of patients would not experience objective rate response (ORR, Figure 7D). A TUNEL quantification has been done on sections following treatments to evaluate the number of tumor apoptotic cells. This technique eliminates the possible biases encountered with the ATP quantifications. The number of apoptotic cells was higher in sections treated with onvansertib (50 nM and 10 nM) as compared to cisplatin (3 μM) and irradiation (2 Gy)-treated sections (Figure 7E). The number of apoptotic cells was higher with the triple combination onvansertib (50 nM), cisplatin (3 μM), 2 Gy as compared to onvansertib alone or cisplatin plus radiotherapy (Figure 7F). The number of apoptotic cells was equivalent to those obtained with onvansertib 100 nM (Figure 7G). Onvansertib, and the combination of onvansertib with the reference treatments also decreased the number of proliferative cells assessed by KI-67 staining (Figure 7H-I). Onvansertib and the triple combination were as effective on primary tumors as on local relapse after cisplatin/radiotherapy.

These experiments represent an upgraded determination of onvansertib relevance on human HNSCC specimens. We suggest its use as a combination treatment with cisplatin/radiotherapy in the first line or alone at relapse. Predetermination of efficacy on diagnostic biopsies may serve to determine the patients eligible for such treatment.

## Discussion

To our knowledge, double resistant HNSCC cells to cisplatin and radiotherapy has never been described. They represent ideal tools to identify mechanisms responsible for the relapses in HNSCC. These cells acquired increased migratory and invasive capacities. Unexpectedly, cisplatin resistant cells acquired resistance to radiotherapy and cells resistant to radiotherapy acquired cisplatin resistance. We can consider the term of “cross resistances” induced by radiotherapy and cisplatin. The intercalating agent properties of cisplatin and the induction of DNA breaks by radiotherapy could explain this phenomenon which depends on DNA damages.

Our objectives were to identify new therapeutic targets to eradicate persistent and/or resistant cells to cisplatin and radiotherapy. Plk1 is a promising target. It is the most investigated member of the Plk family, it plays a central role in the cell cycle 11 and it is overexpressed and associated with a poor prognosis in many cancers including HNSCC 6, 7, melanoma 28, and colon cancer 29 . Hence, we considered Plk1 as an interesting target in these cancers 30. By in silico analysis, we showed that HNSCC express the most Plk1 transcript and its overexpression is associated with a poor prognosis. In the French cohort from Centre Antoine Lacassagne, Plk1 overexpression is higher in patients who relapsed after cisplatin and radiotherapy treatments which strongly suggests an important role during tumor genesis and progression.

Surprisingly, both resistant and sensitive cell lines express the same level of Plk1. Plk1 levels of sensitive cells therefore probably reached a maximal no-modifiable threshold in the resistant cells. This high level is probably due to the low degradation of the protein by the proteasome, a process already described 31. Moreover, Plk1 interacts with proteins essential for the cell cycle. Dysfunction of these interactions could be associated with the overexpression of Plk1 and its capacity to induce tumor development. Bora/Aurora A activates Plk1 at mitosis entry and have an essential role in ovarian tumor development 32. E2F and FoxM1, two transcription factors regulate Plk1 expression 33, 34, thus they could be closely related to Plk1-dependent tumor development 35. Plk1 contributes to carcinogenesis through communication with multiple pathways promoting cancer. Several partners of Plk1 are either tumor suppressor genes or oncogenes. In CAL27 and CAL33, p53 is mutated and inactive. This tumor suppressor is mutated in HNSCC 50% of tumors. Its principal role is to repress Plk1 expression to curb cellular proliferation 36. However, Plk1 phosphorylates and inhibits p53 activity to avoid its anti-proliferative role 37. Non-functional p53 does not repress Plk1 expression, allowing its overexpression and the increase of cell proliferation. Plk1 also interacts with tumor suppressors presented as “guardians of genome”. They are involved in checkpoints like DNA damage checkpoints 38, 39 and spindle assembly checkpoint 40-42 to abrogate cell cycle and protect stability of genome. However, in tumor cells, Plk1 deregulation inhibits the activity of checkpoint actors, maintains cell cycle activity, promotes chromosomal instability and tumorigenesis 43.

The hypomethylation of Plk1 promoter could also explain its overexpression and the aggressiveness of HNSCC cells 44.

In addition of its role in tumorigenesis, Plk1 is involved in EMT which leads to cancer cell dissemination and metastasis through c-RAF-ERK signaling among others 45. Plk1 inhibition using siRNA or pharmacological inhibitors abrogates cancer cell invasion in various cancers like gastric cancer 46.

These arguments are in favor of targeting Plk1 at the diagnosis or after relapse under standard treatments.

Different inhibitors of Plk1 were tested in clinical trials for hematologic and solid tumors, alone or in combination with reference treatments. Rigosertib (ON01910.Na), a non-ATP competitive molecule and a specific inhibitor of Plk1 and PI3K 47, was tested in phase I in patients with advanced solid tumors. An objective response was described for HNSCC patients, but it did not improve the overall survival of patients with pancreatic carcinoma in combination with gemcitabine in phase III 48.

Volasertib is an ATP-competitive and specific inhibitor of Plk1. It has been tested in phase I of advanced solid tumors, in combination with nintedanib. An objective response was obtained and the treatment was well tolerated 49. In a phase III trial in combination with cytarabine, no benefit was described for patients with relapse/refractory acute myeloid leukemia (clinicaltrials.gov, NCT01721876).

Onvansertib is the most specific and selective Plk1 inhibitor. It shows 5000-fold selectivity for Plk1 compared to Plk2/Plk3 16 while Plk1 inhibition by volasertib exhibits only 6- and 65-fold greater selectivity against Plk2 and Plk3 respectively. Rigosertib targets Plk1 and shows only a 30-fold greater selectivity against Plk2, PDGFR, Flt1, BCR-ABL, Fyn, Src, and CDK1 50. These observations confirmed that onvansertib presents a higher specificity for Plk1 as compared to the others Plk1 inhibitors.

Onvansertib is already tested in clinical trial and is now in phase II trials to determine its efficacy on a larger sample of patients. These trials involve patients with untreated acute myeloid leukemia (clinicaltrials.gov, NCT03303339), metastatic colorectal cancer (clinicaltrials.gov, NCT03829410) and metastatic castration-resistant prostate cancer (clinicaltrials.gov, NCT03414034). The patients received onvansertib in combination with reference treatments usually used in each type of cancers.

Despite these clinical trials using onvansertib, HNSCC is the cancer for which the inhibition of Plk1 is the most promising regarding our results (Table S2). Hence, testing onvansertib in clinical trials to increase the lifespan of HNSCC patients represents an important follow-up of our experiments.

Plk1 inhibition results in mitotic catastrophe leading to the decrease of proliferation, cell cycle arrest and apoptosis in HNSCC cells. Onvansertib inhibitor is promising since it has low toxicity on healthy cells and high efficacy on HNSCC sensitive and resistant cells. The inhibition of Plk1 function by onvansertib induced a cell cycle arrest, a defect of mitosis, polyploidy, and apoptosis in resistant and sensitive cells. Onvansertib inhibits their capacities to proliferate, to invade and to form metastases. To complete this *in vitro* study, we also used onvansertib *in vivo*, in two different and complementary models (mice and zebrafish). Tumor growth and metastasis formation were reduced with onvansertib in both models. Plk1 inhibition also induced sensitivity to cisplatin like in ovarian cancer 51 and also to radiotherapy in glioblastoma 52 and osteosarcoma 53. The association of onvansertib and cisplatin and/or radiotherapy, increased cell death meaning that Plk1 inhibition potentiated cisplatin and radiotherapy effects and re-sensitized resistant cell lines to cisplatin and radiotherapy treatments. The association of the three treatments should reduce concentrations of each treatment to avoid toxicity and side effects in patients. Hence, onvansertib could be combined to radiotherapy and cisplatin at the diagnosis (reduce doses to limit toxicity and increased anti-tumor efficacy) but also when patients relapse.

To complete *in vitro* and *in vivo* experiments, onvansertib has been tested on tumors sections of biopsies from HNSCC. It presented a higher efficacy at low concentrations compared to cisplatin and radiotherapy. By combining onvansertib with cisplatin and radiotherapy, its concentration has been divided by two and its efficacy was improved. This association re-sensitize to cisplatin and radiotherapy all tumors that are resistant to these treatment (60% of HNSCC patients tested have already have cisplatin/radiotherapy treatment and relapse). In patients, reduced concentrations of each treatment should decrease their toxicity and side effects while maintaining high efficiency. Moreover, this triple association is as efficient in primary tumors as in local relapse. Thus, onvansertib could be used as a first-line treatment in combination with cisplatin and radiotherapy to increase their efficacy and to prevent acquired resistance but also when patients relapse to cisplatin/radiotherapy.

This study represents a proof of concept to initiate clinical trials using onvansertib for patient in therapeutic failure or in the first line in combination with cisplatin and radiotherapy. Based on our results we can expect spectacular responses with increase progression free or overall survival.

## Supplementary Material

Supplementary figures and tables.
