# Supplementary material for: The Polo-like kinase 1 inhibitor onvansertib represents a relevant treatment for head and neck squamous cell carcinoma resistant to cisplatin and radiotherapy
Source: Theranostics. 2021 Sep 21;11(19):9571–86. doi: 10.7150/thno.61711 (PMC8490521; doi:10.7150/thno.61711)
Supplement: Supplementary file 1 — Supplementary figures and tables. [file thnov11p9571s1.pdf]

Figure S1. Impact of Plk1 expression on lymph node invasion and HPV status. (**A**) Analysis of the levels of Plk1 mRNA in HNSCC patients with (N1) or without (N0) lymph node metastasis. (**B**) Analysis of the levels of Plk1 mRNA in HNSCC patients with or without (N0) HPV infection.

Figure S2. Characterization of CAL27 sensitive and resistant cell lines. (**A**) Plk1 Immunoblotting realized on CAL33 and CAL27 sensitive and resistant cell lines. GAPDH and ARD1 served as loading controls. (**B**) Clonogenicity assays of CAL27, CAL27 cis-R and CAL27 RR after 4 Gy of radiation (n = 2). (**C**) Cell viability evaluated by XTT assays on CAL27, CAL27 cis-R and CAL27 RR after 48 h of cisplatin treatment (n = 3). (**D-E**) Cell migration using Boyden's chamber assay on CAL27 sensitive and resistant cell lines (**D**). Results are expressed as the percentage of the control (n = 3) (**E**). (**F-G**) Spheroids' invasion using 3D culture cell assays on sensitive and resistance CAL27 cells (**F**). Results are expressed as arbitrary units (day 0 used as the control) (n = 2) (**G**). Statistics were performed using *ANOVA test*: * $p < 0.05$, ** $p < 0.01$, *** $p < 0.001$, **** $p < 0.0001$.

Figure S3. Specificity and efficacity of onvansertib. (**A**) Onvansertib inhibits the phosphorylation of two Plk1 substrates, TCTP and Cdc25C. The phosphorylation of TCTP and Cdc25C in CAL27RR (left) and CAL33RR (right) was determined by immunoblots. GAPDH served as a loading control. (**B**) Quantification of the number of colonies in naive, Cis-R, Rad-R, and RR CAL33 and CAL27 cells treated with increasing doses of onvansertib, cisplatin and radiotherapy. (**C**) Clonogenicity assays on sensitive and resistant CAL33 and CAL27 cells treated with increasing doses of onvansertib, cisplatin and radiotherapy. Statistics were performed using *ANOVA test*: * $p < 0.05$; ** $p < 0.01$; *** $p <0.001$; **** $p < 0.0001$.

Figure S4. Onvansertib effects on sensitive and resistant CAL27 cells. (**A**) Clonogenicity assays on sensitive and resistant CAL27 cells after treatment by onvansertib (10 nM, 25 nM) or cisplatin (3 µM) (n = 2). (**B**-**C**) CAL27 cells were treated with different concentrations of onvansertib (10 nM, 25 nM, 50 nM) for 24 h. Cell cycle was measured by flow cytometry (n = 3) (**B**). G2/M arrest was evaluated by phospho-NPM and, phospho-H3 immunoblotting. HSP90 served as a loading control (n = 2) (**C**). (**D-E**) Polyploidy (number of nuclei 8N and 16N) was assessed by flow cytometry (**D**). Representation of polyploidy in CAL27 RR (n = 3) (**E**). (**F**) Cells were treated with onvansertib (25 nM, 50 nM) for 48 h. Cell death was evaluated by flow cytometry. Cells were stained with PI and Annexin V. Histograms show AV^{+}/PI^{-} cells (apoptosis) and AV^{+}/PI^{+} cells (post-apoptosis or another cell death) (n = 3). (**G**) Immunostaining of PARP expression on sensitive and resistant CAL27 cells after an exposure to different concentrations of onvansertib (100 nM) for 24h. HSP60 antibody was used as a loading control (n = 2). (**H**) Apoptosis mediated by siRNA dependent inhibition of Plk1 expression evaluated by annexin V and propidium iodide staining after 96 h and 120 h. Effects of Plk1-directed siRNA on Plk1 expression by immunoblot after 48 h. GAPDH is shown as a loading control. Statistics were performed using Student's *t test:* *** p < 0.001 (n = 3). (**I**) Spheroid invasion using 3D cell culture assay on sensitive and resistant CAL27 cells after 6 days of onvansertib treatment (100 nM). Results are represented as arbitrary units (CT condition used as the reference 100%) (n = 2). Statistics were performed using *ANOVA test*: * $p < 0.05$, ** $p < 0.01$, *** $p < 0.001$, **** $p < 0.0001$.

Figure S5. Efficacity of onvansertib alone or in combination with cisplatin or radiotherapy. (**A-D**) Clonogenicity assays realized on sensitive and resistant CAL33 and CAL27 cells treated with onvansertib (5 nM) in combination with radiotherapy (2

Gy) (**A-B**) or in combination with cisplatin (1 µM) (n = 3) (**C-D**). (**E**) Analyzes of synergic effects of onvansertib (2.5 nM or 5 nM) in combination with cisplatin (1 µM) or radiotherapy (2 Gy) or both (n = 3).

Figure S6. Onvansertib effects in combination with reference treatments (cisplatin and radiotherapy) on sensitive and resistant CAL27 cells. (**A**) CAL27 cells were treated with onvansertib (25 nM) in combination with cisplatin (1 µM) and radiotherapy (8 Gy) for 72 h. Cell death was evaluated by flow cytometry (PI and Annexin V). Histograms show AV^+/PI^- cells (apoptosis) and AV^+/PI^+ cells (post-apoptosis or another cell death). (**B-C**) Cells were treated with onvansertib (2.5 nM), cisplatin (1 µM) and radiotherapy (2 Gy). Representative images (**B**) and quantification (**C**) of clonogenicity assays. (**D-E**) Spheroids were treated with onvansertib (25 nM), cisplatin (3 µM) and radiotherapy (2 Gy) for 6 days. Results are represented as arbitrary units (no treatment condition used as the reference 100%) (n = 2). Statistics were performed using *ANOVA test*: * p < 0.05, ** p < 0.01, *** p < 0.001, **** p < 0.0001 and unpaired Student's *t test*: * p < 0.05, ** p < 0.01, **** p < 0.0001.

Figure S7. onvansertib effects in combination with reference treatments (cisplatin and radiotherapy) on healthy tissues. ATP quantification of healthy tissues treated with onvansertib (100 nM), cisplatin (10 µM) and radiotherapy (2 Gy) alone or in combination.

Table S1. Characteristics of the patients included in the study (n = 20).

Table S2. IC50 for cell viability (XTT assays) under onvansertib on different cancer cell lines.

Table S3. Clinical parameters of HNSCC patients from which biopsies were obtained (n = 6).

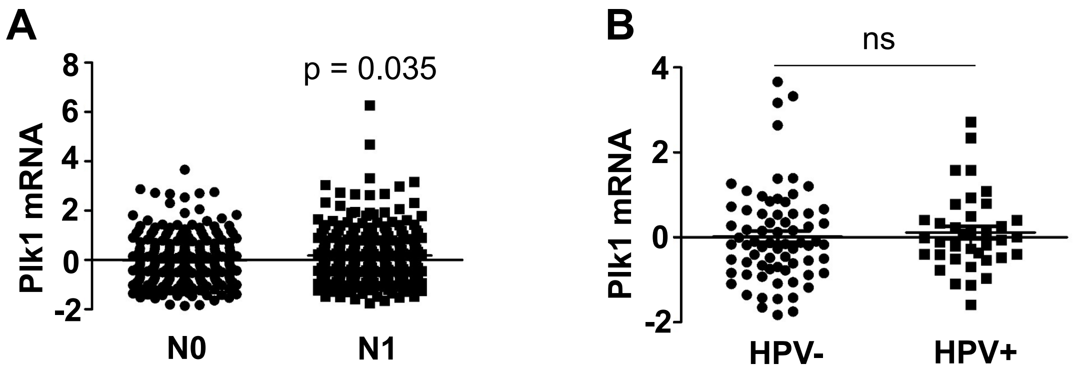
A
Plk1 mRNA
8
6
4
2
0
-2
p = 0.035
N0
N1
B
ns
Plk1 mRNA
4
2
0
-2
HPV-
HPV+

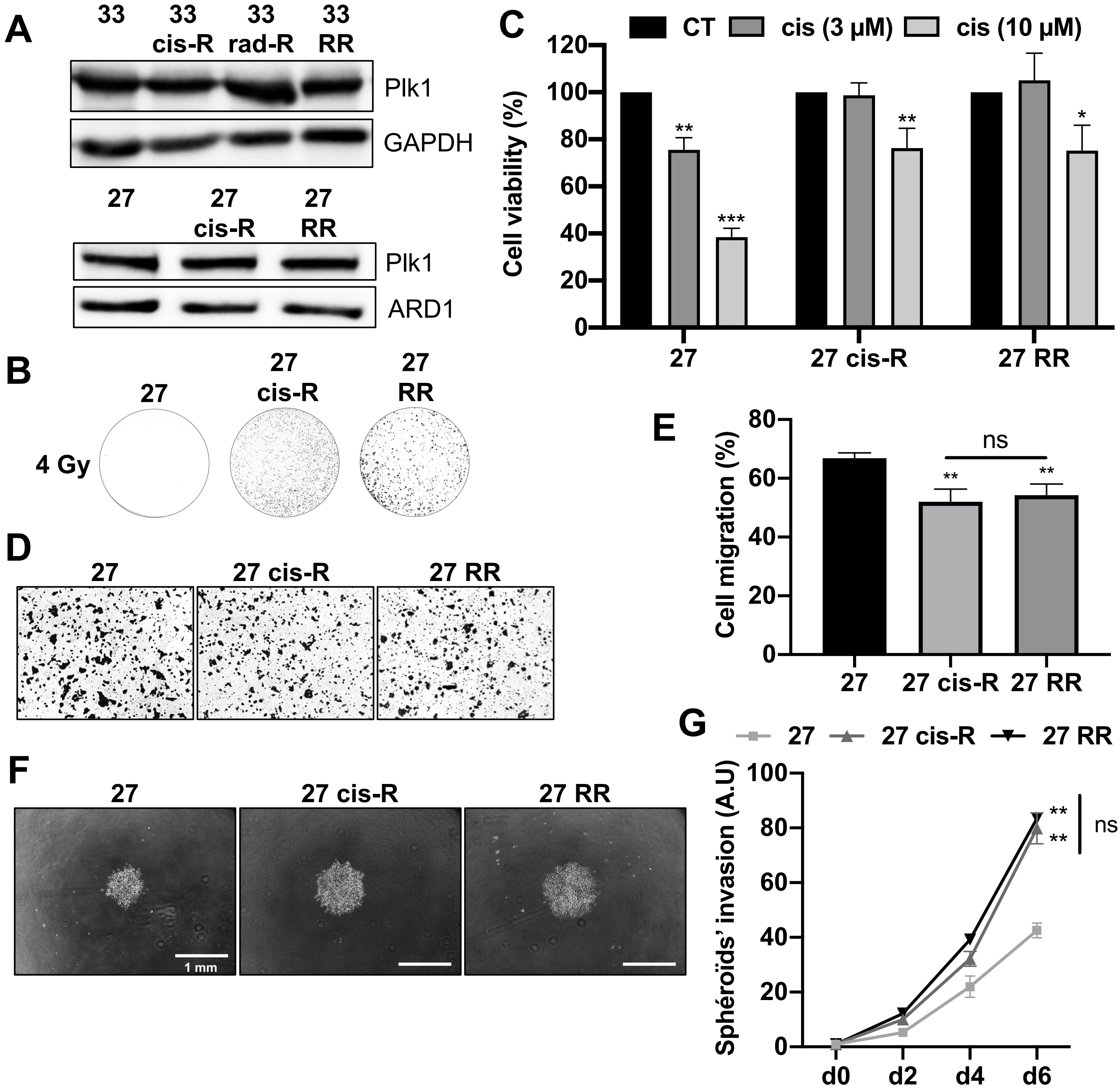

A
33
33 cis-R
33 rad-R
33 RR
Plk1
GAPDH
27
27 cis-R
27 RR
Plk1
ARD1
B
27
27 cis-R
27 RR
4 Gy
C
CT
cis (3 µM)
cis (10 µM)
Cell viability (%)
0
20
40
60
80
100
120
**
***
**
*
27
27 cis-R
27 RR
D
27
27 cis-R
27 RR
E
Cell migration (%)
0
20
40
60
80
ns
**
**
27
27 cis-R
27 RR
F
27
27 cis-R
27 RR
1 mm
G
27
27 cis-R
27 RR
Sphéroïds' invasion (A.U)
0
20
40
60
80
100
**
**
ns
d0
d2
d4
d6

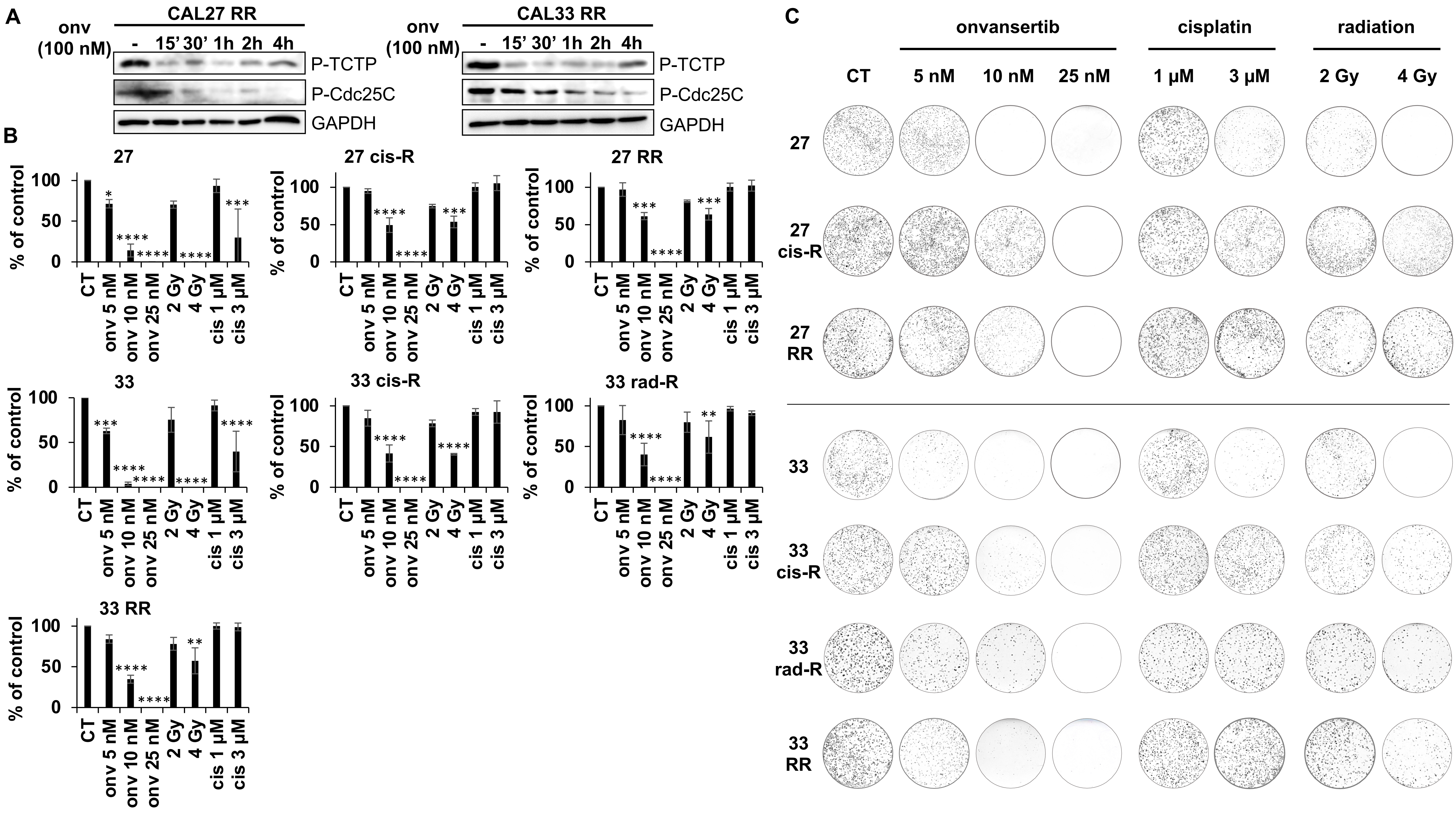

A
onv (100 nM)
CAL27 RR
- 15' 30' 1h 2h 4h
P-TCTP
P-Cdc25C
GAPDH
CAL33 RR
B
27
27 cis-R
27 RR
33
33 cis-R
33 rad-R
33 RR
% of control
CT
onv 5 nM
onv 10 nM
onv 25 nM
2 Gy
4 Gy
cis 1 µM
cis 3 µM
C
onvansertib
cisplatin
radiation
CT
5 nM
10 nM
25 nM
1 µM
3 µM
2 Gy
4 Gy
27
27 cis-R
27 RR
33
33 cis-R
33 rad-R
33 RR

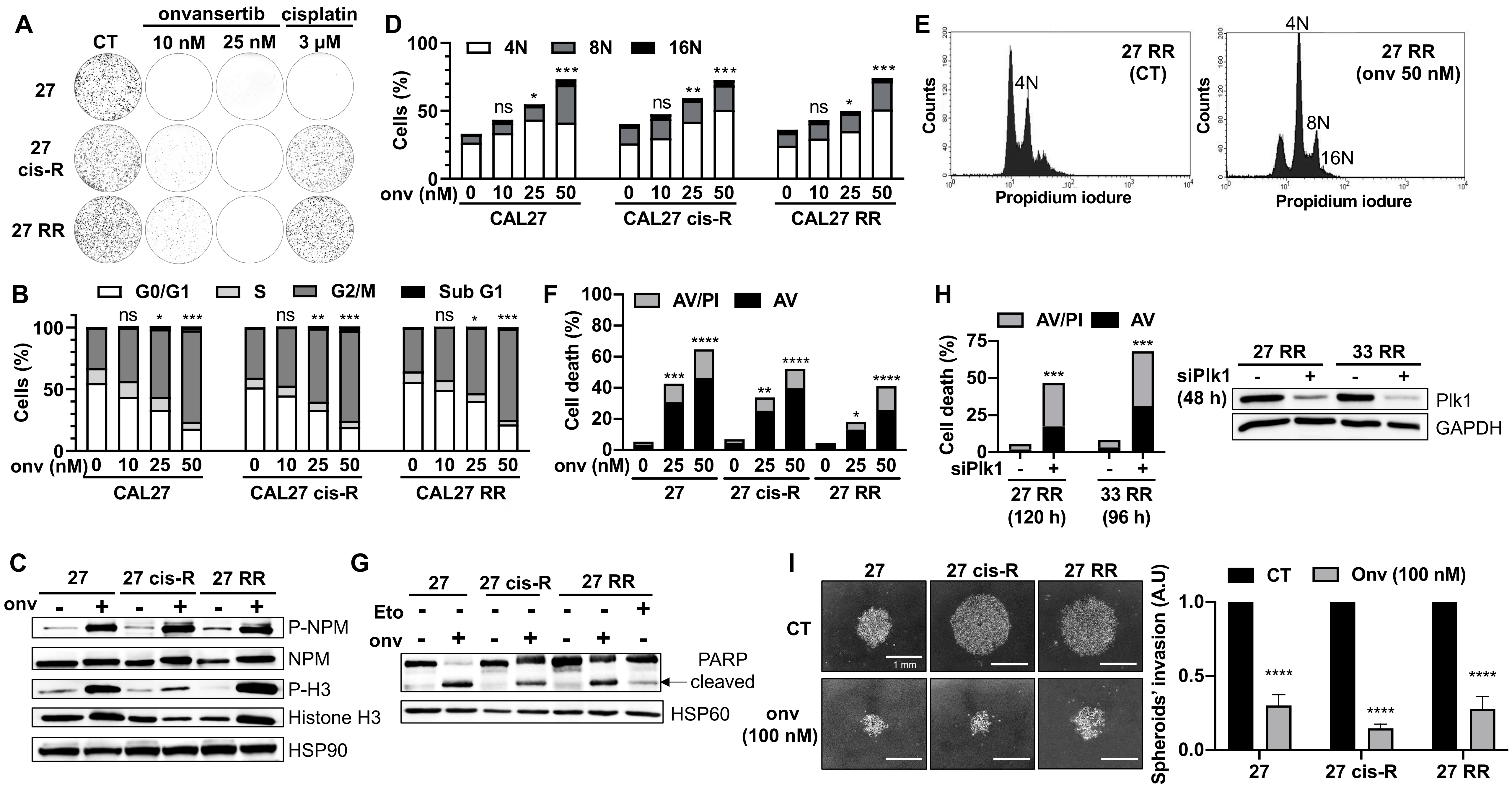

A
onvansertib
cisplatin
CT
10 nM
25 nM
3 µM
27
27 cis-R
27 RR
D
4N
8N
16N
Cells (%)
onv (nM)
CAL27
CAL27 cis-R
CAL27 RR
E
27 RR (CT)
27 RR (onv 50 nM)
Counts
Propidium iodure
4N
8N
16N
B
G0/G1
S
G2/M
Sub G1
Cells (%)
onv (nM)
CAL27
CAL27 cis-R
CAL27 RR
F
AV/PI
AV
Cell death (%)
onv (nM)
27
27 cis-R
27 RR
H
AV/PI
AV
Cell death (%)
siPlk1
27 RR (120 h)
33 RR (96 h)
27 RR
33 RR
siPlk1 (48 h)
Plk1
GAPDH
C
27
27 cis-R
27 RR
onv
P-NPM
NPM
P-H3
Histone H3
HSP90
G
27
27 cis-R
27 RR
Eto
onv
PARP
cleaved
HSP60
I
27
27 cis-R
27 RR
CT
onv (100 nM)
1 mm
CT
Onv (100 nM)
Spheroids' invasion (A.U)
27
27 cis-R
27 RR

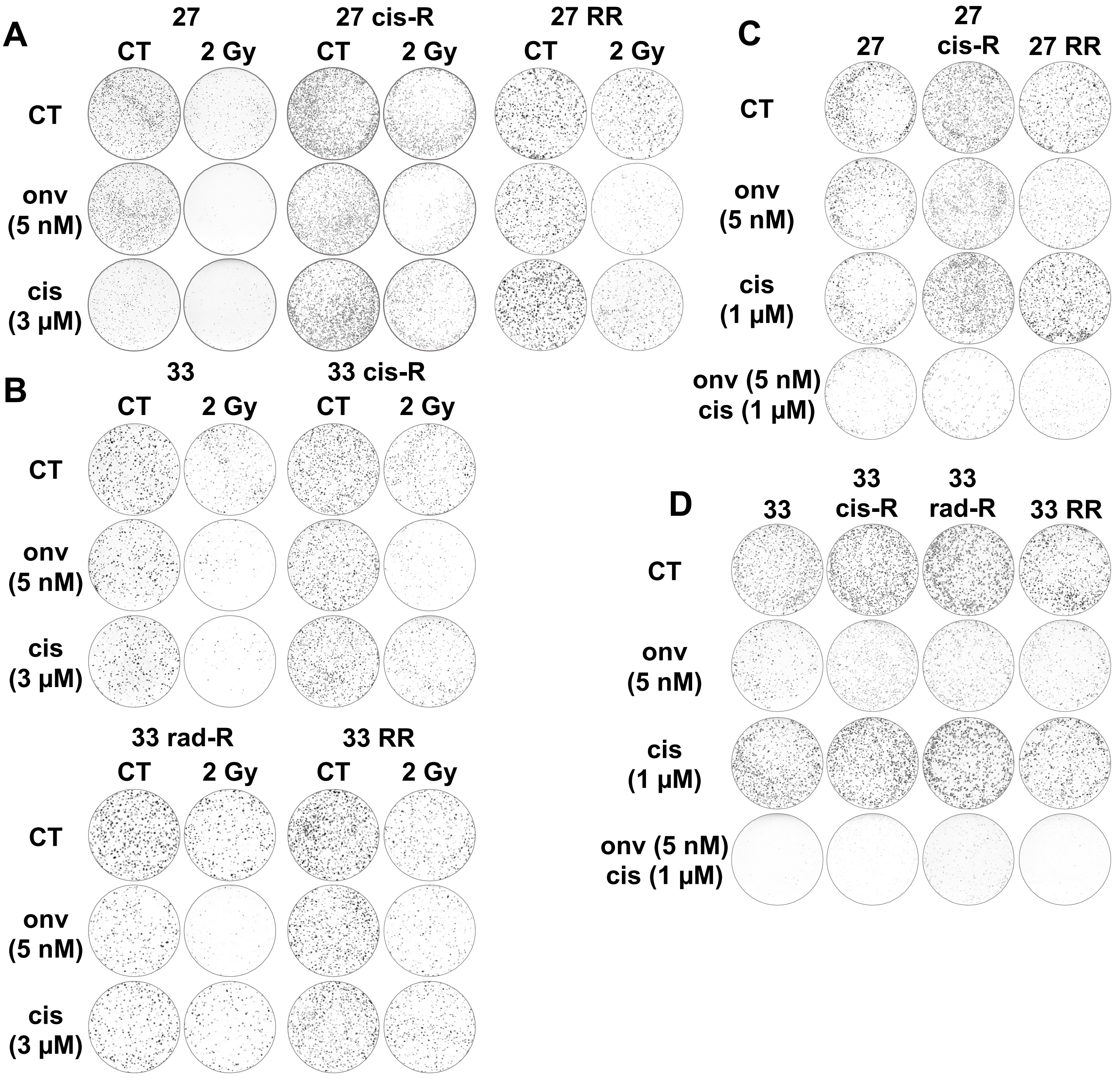

E		cis (1 µM)	2 Gy	onv (2.5 nM)	onv (5 nM)	cis (1 µM) + 2 Gy	onv (5 nM) + 2 Gy	onv (5 nM) + cis (1 µM)	onv (2.5 nM) + cis (1 µM) + 2 Gy
**CAL27**	Estimated value					-7	-19	-14	-20
	True value	0	0.27	3.1	1.3	-12	-38	-40	-41
	**p value**					**ns**	**0.02**	**0.005**	**0.0014**
**CAL27 cis-R**	Estimated value					-28	-40	-23	-31
	True value	0	-25	-1.7	-5	-28	-74	-58	-68
	**p value**					**ns**	**2.90E-05**	**4.60E-04**	**7.90E-05**
**CAL27 RR**	Estimated value					-19	-39	-8	-30
	True value	0	-18	-1.8	-3.2	-21	-67	-38	-61
	**p value**					**ns**	**8.00E-05**	**0.012**	**2.6E-04**
**CAL33**	Estimated value					-18	-23	-21	-28
	True value	0	-16	8	-28	-23	-62	-60	-69
	**p value**					**0.069**	**1.00E-07**	**1.00E-07**	**1.00E-07**
**CAL33 cis-R**	Estimated value					-10	-27	-13	-28
	True value	0	-14	-2	-8	-17	-51	-30	-54
	**p value**					**0.03**	**1.00E-07**	**1.40E-04**	**1.00E-07**
**CAL33 rad-R**	Estimated value					-16	-34	-30	-26
	True value	0	-5	5	-14	-18	-62	-58	-56
	**p value**					**ns**	**9.00E-06**	**2.30E-05**	**3.10E-05**
**CAL33 RR**	Estimated value					-10	-25	-14	-32
	True value	0	-12	-2	-16	-21	-53	-44	-63
	**p value**					**0.016**	**1.00E-07**	**2.00E-06**	**1.00E-07**

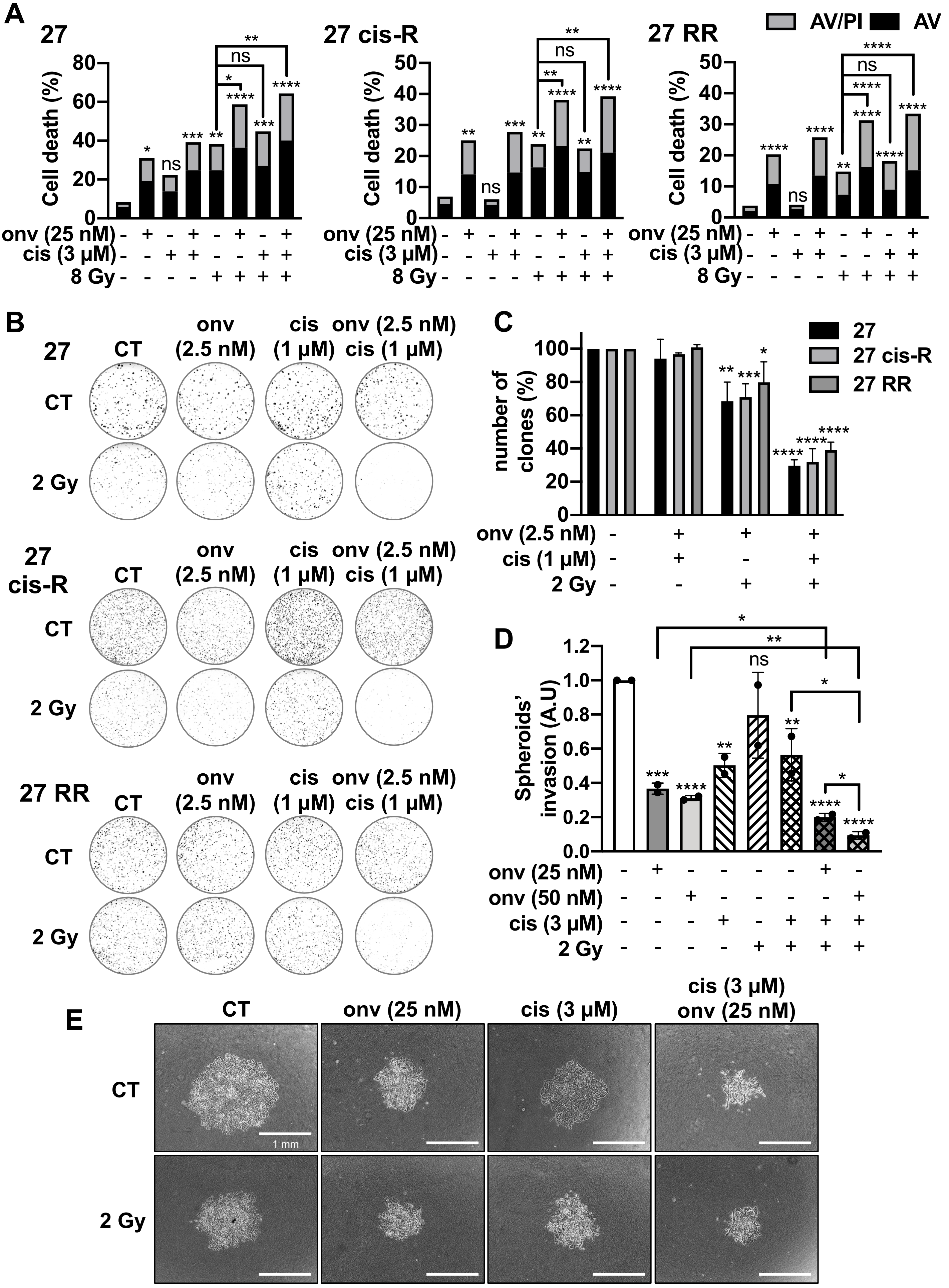
A
27
27 cis-R
27 RR
AV/PI
AV
Cell death (%)
onv (25 nM)
cis (3 μM)
8 Gy
B
27
27 cis-R
27 RR
CT
onv (2.5 nM)
cis (1 μM)
onv (2.5 nM) cis (1 μM)
2 Gy
C
number of clones (%)
onv (2.5 nM)
cis (1 μM)
2 Gy
D
Spheroids' invasion (A.U)
onv (25 nM)
onv (50 nM)
cis (3 μM)
2 Gy
E
CT
onv (25 nM)
cis (3 μM)
cis (3 μM) onv (25 nM)
2 Gy
1 mm

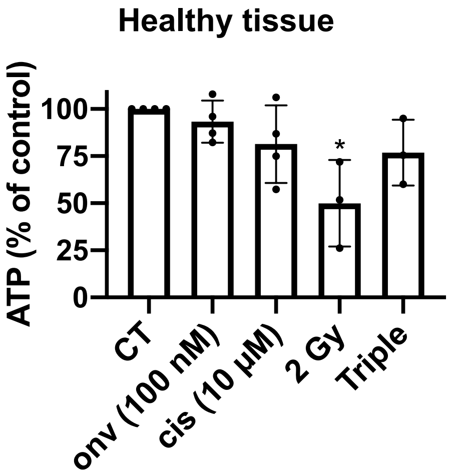
Healthy tissue
ATP (% of control)
100
75
50
25
0
*
CT
onv (100 nM)
cis (10 μM)
2 Gy
Triple

HNSCC	Diagnostic	Relapse after radiotherapy + platin treatment
**Number**	20	
**Sex**		
Female	5 (15%)	
Male	15 (75%)	
**pT**		
1/2	7 (77.8%)	6 (54.5%)
3/4	2 (22.2%)	5 (45.5%)
x	11	9
**pN**		
0	3 (33.3%)	10 (90.9%)
≥ 1	6 (66.7%)	1 (9.1%)
x	11	9
**pM**		
0	20 (100%)	16 (80%)
1	0 (0%)	4 (20%)
**Location**		
Oral cavity	10 (50%)	5 (25%)
Oropharynx	7 (35%)	13 (65%)
Larynx	3 (15%)	0 (0%)
Lung	0 (0%)	2 (10%)
**DFS (median)**	37 months	
**OS (median)**	119.1 months	

**cell lines**	**cancer type**	**onvansertib IC50 (nM)**
FHN (fibroblasts)	healthy	> 250
keratynocytes	healthy	> 250
BT-549	Carcinoma breast ductal	150
**Detroit 562**	**Squamous cell carcinoma**	**25**
**CAL33**	**Squamous cell carcinoma**	**25**
**CAL33 cis-R**	**Squamous cell carcinoma**	**25**
**CAL33 rad-R**	**Squamous cell carcinoma**	**25**
**CAL33 RR**	**Squamous cell carcinoma**	**25**
**CAL27**	**Squamous cell carcinoma**	**25**
**CAL27 cis-R**	**Squamous cell carcinoma**	**25-50**
**CAL27 RR**	**Squamous cell carcinoma**	**25-50**
NB4	Acute promyelocytic leukemia (LAM)	100
U937	Histiocytic lymphoma	50
U266	Multiple Myeloma	50
8226	Multiple Myeloma	100
786	Renal cells carcinoma (RCC)	125
Mel202	Uveal melanoma	125
DU-145	Prostate cancer	250
MDA-MB-231	Breast carcinoma	200

HNSCC patients	
**Number**	6
**Tumor from**	
Diagnostic	3 (50%)
Relapse after after radiotherapy + platin treatment	3 (50%)
**Sex**	
Female	3 (50%)
Male	3 (50%)
**pT**	
1/2	1 (20%)
3/4	5 (80%)
**pN**	
0	3 (70%)
≥ 1	2 (30%)
x	1
**pM**	
0	5 (80%)
1	1 (20%)
**Location**	
Oral cavity	4 (70%)
Oropharynx	2 (30%)
